# Don’t get it or don’t spread it: comparing self-interested versus prosocial motivations for COVID-19 prevention behaviors

**DOI:** 10.1038/s41598-021-97617-5

**Published:** 2021-10-12

**Authors:** Jillian J. Jordan, Erez Yoeli, David G. Rand

**Affiliations:** 1 Harvard Business School, Boston, USA; 2 Sloan School of Management, Massachusetts Institute of Technology, Cambridge, USA

**Keywords:** Psychology, Human behaviour

## Abstract

COVID-19 prevention behaviors may be seen as self-interested or prosocial. Using American samples from MTurk and Prolific (total *n* = 6850), we investigated which framing is more effective—and motivation is stronger—for fostering prevention behavior intentions. We evaluated messaging that emphasized *personal*, *public*, or *personal and public* benefits of prevention. In initial studies (conducted March 14–16, 2020), the Public treatment was more effective than the Personal treatment, and no less effective than the Personal + Public treatment. In additional studies (conducted April 17–30, 2020), all three treatments were similarly effective. Across all these studies, the perceived *public* threat of coronavirus was also more strongly associated with prevention intentions than the perceived *personal* threat. Furthermore, people who behaved prosocially in incentivized economic games years before the pandemic had greater prevention intentions. Finally, in a field experiment (conducted December 21–23, 2020), we used our three messaging strategies to motivate contact-tracing app signups (*n* = 152,556 newsletter subscribers). The design of this experiment prevents strong causal inference; however, the results provide suggestive evidence that the Personal + Public treatment may have been more effective than the Personal or Public treatment. Together, our results highlight the importance of prosocial motives for COVID-19 prevention.

## Introduction

The COVID-19 pandemic has caused millions of deaths worldwide, and continues to pose an enormous global threat. It thus remains essential that people engage in behaviors that help prevent infection (e.g., masking, personal hygiene, and physical distancing)^1^ even after the initial introduction of vaccines^2^. Many preventative behaviors, however, are difficult to adhere to or require substantial personal sacrifices. What motivates people to engage in them?

One reason this question is interesting is that coronavirus prevention behaviors protect both the individuals who engage in them and society at large. Thus, they may be conceptualized as either self-interested actions (that serve to benefit the actor) or as cooperative efforts (that, in addition to benefiting the actor, serve also to benefit others in society). Here we investigate the relative strength of these motivations, and the relative efficacy of these framings, for encouraging prevention behaviors.

One might naturally expect a dominant role of self-interest for coronavirus prevention. According to classical economic theories of decision-making, people care only for their own welfare. This perspective would suggest that self-interest should be the strongest motivator. As Adam Smith famously wrote, “It is not from the benevolence of the butcher, the brewer, or the baker that we expect our dinner, but from their regard to their own interest”^3^.

Yet while the classical economic perspective remains highly influential, behavioral scientists are increasingly aware of the importance of more psychologically informed theories^4–7^. Research in psychology and behavioral economics provides clear evidence that people care about the welfare of others and are motivated to cooperate^8–12^, and that people strive to avoid appearing selfish in the eyes of others and are highly sensitive to social norms^13–16^. Thus, psychological research reveals that people have “prosocial motivations” (a term we use to refer to any motivation to promote the welfare of others—including those that may be implicitly or explicitly driven by reputation concerns or the desire to adhere to social norms).

While much of this research—which has focused extensively on behavior in economic games—suggests that people care more about their own welfare than the welfare of others, regard for others seems especially strong in the domain of physically aversive outcomes. Experiments reveal that people sometimes voluntarily undergo painful experiences to relieve the suffering of others^17^. Furthermore, people tend to be more risk-averse when making decisions on behalf of others^18^, including in the context of physical harm^19^. And evidence suggests that when people are tasked with allocating *pain* between themselves versus others, they tend to behave more generously than when they are tasked with allocating *money* between themselves versus others^20–22^.

In one such study, people were actually less willing to harm others than to harm themselves^23^. However, other studies have demonstrated that, even in the domain of harm, people prioritize their own outcomes equally or above the outcomes of others^20–22^. Thus, previous basic science research investigating prosociality provides some reason to doubt that self-interested motives are dominant, and self-interested framings are more effective, for coronavirus prevention. Yet it nonetheless provides no clear prediction regarding the relative role of prosociality.

Another stream of applied research has investigated self-interested versus prosocial motives in the context of health behavior (and especially vaccination decisions), and has provided clear evidence that both motives can encourage disease prevention^24–28^. A few studies have directly compared the *relative* effectiveness of personal versus public framing in vaccination appeals, with inconsistent results: some studies found that public framing was ineffective^29–31^, while others provided some mixed evidence for the effectiveness of public framing^32,33^; and a set of field studies investigating handwashing among healthcare professionals found that it was more effective to emphasize patient safety than personal safety^34^. Thus, the literature surrounding disease spread prevention likewise does not make a clear prediction regarding the relative importance of self-interested versus prosocial motivations, and the relative efficacy of self-interested versus prosocial framing, for coronavirus prevention.

To investigate, we measure the influence of three messaging treatments on intentions to engage in COVID-19 prevention behaviors: one that emphasizes *personal* benefits of prevention (Personal message), one that emphasizes *public* benefits of prevention (Public message), and one that emphasizes both types of benefits (Personal + Public message). Our *Personal* message was designed to encourage subjects to simply consider the value of prevention behaviors for themselves, while our *Public* message was designed to prompt subjects to consider that prevention behaviors can, in addition to conferring personal benefits, also benefit others. Finally, our Personal + Public message was designed to explicitly encourage subjects to consider both types of benefits.

In a first set of studies (Studies 1–2, total *n* = 2176) conducted early in the pandemic (between March 14 and March 16, 2020, at which time there were under 2000 confirmed U.S. cases), we find that (i) the Public message was more effective than the Personal message, and (ii) the Personal + Public message was no more effective than the Public message. In a second a set of studies (Study 3a–d, total *n* = 3985) conducted slightly later in the pandemic (between April 17 and April 30, 2020, at which time there were 500,000 to 1,000,000 confirmed U.S. cases), we find that all three messaging strategies were similarly effective.

We also take a correlational approach to investigate the extent to which prosocial versus self-interested motivations predict prevention intentions. Across Studies 1–3, as well as an additional study using a more representative sample (Study 4), we consistently find that the perceived *public* threat of coronavirus is a stronger predictor of prevention intentions than the perceived *personal* threat. And in Study 5, by linking data from Studies 1–3 to an external dataset of incentivized economic game decisions, we find that people who behaved prosocially years before the pandemic report greater prevention intentions.

Finally, in Study 6, which we conducted more than half a year later (between December 21 and December 23, 2020), we employ a field experiment to investigate the power of our three aforementioned messaging strategies to motivate people to sign up for a contact tracing app. Randomization in the experiment was imperfect, preventing strong causal inference, but the results provide suggestive evidence that combining self-interested and prosocial framing may have been more effective than self-interested or prosocial framing alone.

Together, our results challenge the hypotheses that self-interested motives are the dominant driver of prevention intentions and self-interested appeals are the most effective messaging strategy. Instead, they suggest that prosociality can play an important role in combatting COVID-19.

## Studies 1 and 2

We begin by describing the method and results of our first set of studies, conducted in the early days of the COVID-19 pandemic reaching the United States.

For all studies reported in this paper, written informed consent was obtained from all participants, the study was approved by the Massachusetts Institute of Technology Committee on the Use of Humans as Experimental Subjects, and all research was performed in accordance with relevant guidelines and regulations.

## Methods

### Overview

On March 14 (Study 1) and March 16 (Study 2), 2020, we conducted two studies online using convenience samples from Amazon Mechanical Turk (Mturk). Although there is a considerable amount of evidence supporting the validity of Mturk samples for social science research^35,36^, Mturk samples are not nationally representative. Most notably, participants from Mturk skew much younger than the national age distribution. Yet we nonetheless see our Mturk samples as representative of an important population. Young people are a frequent target for COVID-19 prevention messaging, because they are perceived as less likely to engage in prevention behaviors (a perception that is consistent with our data from all studies in this paper; see Supplementary Sect. 4.1).

Studies 1–3 were all pre-registered, and our sample sizes were based on our pre-registrations. Furthermore, our analyses adhere closely to our pre-registered analysis plans. We note the substantive exceptions in our main text where relevant, and list all exceptions (as well as links to our pre-registrations) in Supplementary Sect. 5. We also note that for all studies in this paper, our full materials, raw data, and a script reproducing all analyses are available at https://osf.io/sr4n9/.

Studies 1 and 2 used very similar designs, but differed in a few ways. We begin by describing Study 1, and then describe the ways that Study 2 differed from Study 1.

### Design

In Study 1, we recruited a target of *n* = 1000 subjects, and assigned them to one of four experimental conditions, which included a control condition (involving no treatment) and three treatment conditions (Personal, Public, and Personal + Public).

After obtaining consent from all subjects, we presented subjects in our treatment conditions with their assigned treatment. In all three treatment conditions, subjects were presented with (i) some written text and then (ii) a flier, before being directed to complete our set of outcome measures. In contrast, in the control condition, subjects were not presented with a written text or a flier, and began the study by completing our set of outcome measures.

We designed our three treatment conditions to be very similar, but manipulated whether they emphasized the *personal*, *public,* or *personal and public* benefits of coronavirus prevention behaviors. We note that while we see our manipulations (both in Study 1 and throughout this paper) as having high face validity, we did not collect any manipulation check measures to evaluate whether these benefits were successfully communicated to subjects.

### Treatments

In all treatments, we first assigned subjects to read some written text about COVID-19, and then presented subjects with a flier about COVID-19.

#### Written text

To introduce the written text portion of our treatments, we presented subjects with the following text: “Please read the following information about COVID-19, which the World Health Organization has recently classified as a pandemic.” We then began by providing subjects with three paragraphs of basic information about coronavirus and the threat it poses. This portion of the written text was identical across treatments; see Supplementary Sect. 6 for text. Next, we encouraged subjects to take the virus seriously and take preventative action. This portion of the written text varied across treatments. In the *Personal* treatment, it read:

For all of these reasons, **coronavirus is a serious threat to you**. It is recommended that you take this threat very seriously to prevent contracting COVID-19 and getting very ill or dying. Fortunately, **there are steps you can take to keep yourself safe.**

In the *Public* treatment, it read:

This means **coronavirus is a serious threat to your community**. It is recommended that you take this threat very seriously to prevent spreading COVID-19 and causing people in your community to get very ill or die. Fortunately, **there are steps you can take to keep your community safe.**

And in the *Personal* + *Public* treatment, it read:

This means **coronavirus is a serious threat to you and your community**. It is recommended that you take this threat very seriously to prevent contracting COVID-19 and getting very ill or dying, or spreading COVID-19 and causing people in your community to get very ill or die. Fortunately, **there are steps you can take to keep yourself and your community safe.**

Finally, the written text concluded by presenting a final paragraph encouraging subjects to engage in prevention behaviors. This portion of the text was constant across treatments; see Supplementary Sect. 6 for text.

Thus, in the Personal treatment, we emphasized the threat to the subject, in the Public treatment, we emphasized the threat to the subject’s community, and in the Personal + Public treatment, we emphasized the threat to the subject and their community. We note that the difference between the clause “for all these reasons” (Personal treatment) and “this means” (Public treatment and Public + Personal treatment) reflects an unintentional error; however, we believe that it is very unlikely to account for our results.

Furthermore, we note that our treatments varied slightly in length. However, we similarly believe that length differences are unlikely to account for our results. The length differences across treatments were quite minimal—and, as noted above, all treatments were accompanied by four paragraphs of text that was constant across treatments (and is reported in the Supplementary Information). Thus, the percentage difference in word length across treatments is very small.

#### Fliers

**Figure 1:**
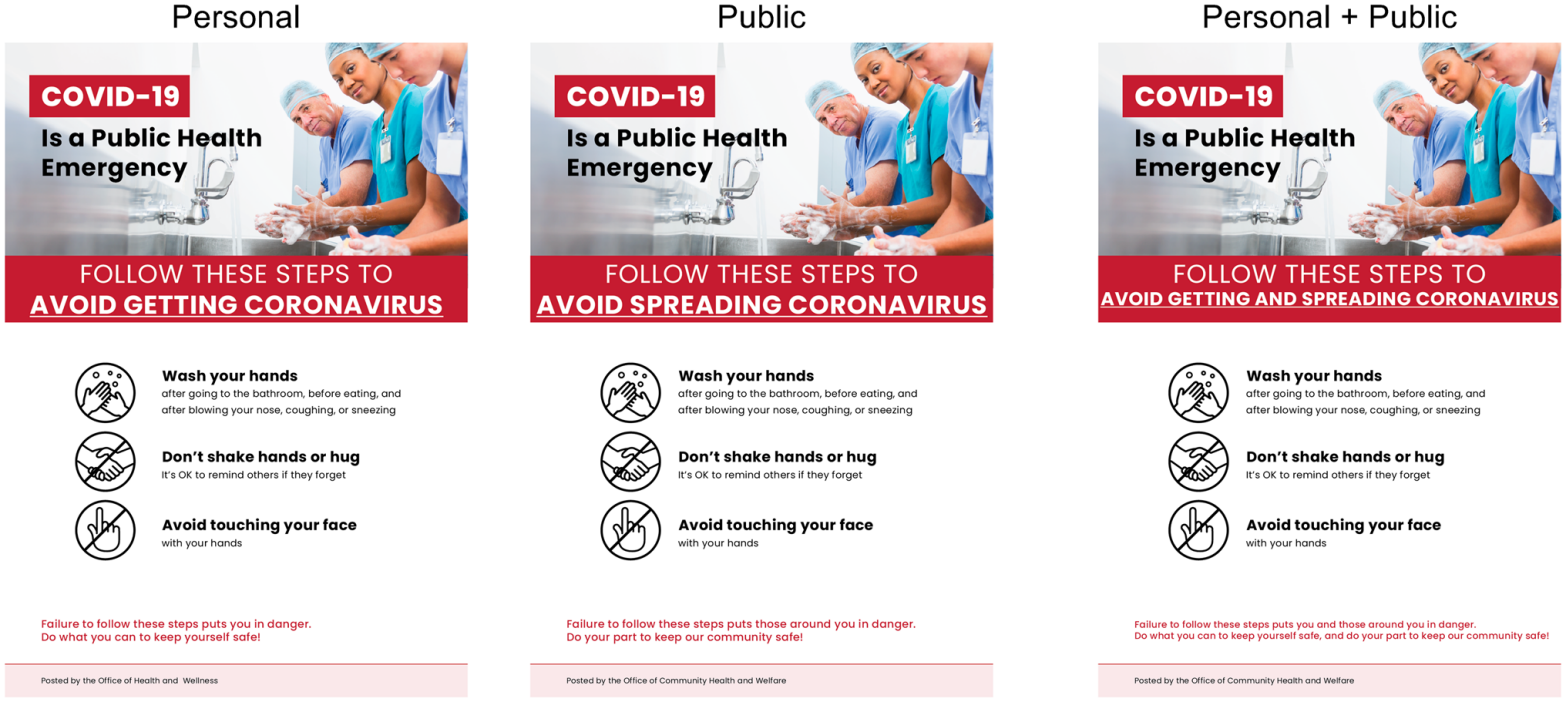
Fliers shown in each treatment. Photograph attribution: iStock.com/kali9.

After subjects finished reading this text, they were asked to carefully read a flier about COVID-19 (see Fig. 1). To introduce the flier portion of our treatments, we presented subjects with the following text: “Thank you. Now, please carefully look at this flier, which provides further information about COVID-19.” We then displayed a flier that varied across treatments, again by emphasizing threat to the subject, their community, or both. The images in the fliers were purchased from the stock photo provider istockphoto.com.

### Measures

After the treatments were presented to subjects in the treatment conditions, all subjects completed a series of outcome measures. We began by measuring (i) our two dependent variables and (ii) the perceived public and personal threat of coronavirus, manipulating between-subjects which set of variables we measured first. Finally, we concluded by measuring a set of individual difference variables, described in Supplementary Sect. 1.

#### Dependent variables

We collected two key dependent variables in a fixed order.

First, subjects reported their *prevention intentions.* To do so, they reported their intentions, on 0–100 sliding scales, to engage in a series of 11 prevention behaviors (“wash my hands at least 10 times a day”, “wash my hands more often”, “stop shaking other people’s hands”, “stop hugging people”, “try my hardest to avoid touching my face”, “stay home if I am feeling even a little bit sick”, “try to stay home whenever possible, even if I am not sick”, “cover my mouth when I cough and sneeze”, “purchase food reserves and medication”, and “stock up on cleaning supplies”). To create a composite measure of prevention intentions, we averaged intentions to engage in these 11 behaviors.

Next, subjects reported a more detailed set of *social distancing intentions.* While our above-described set of questions about prevention intentions included an overall social distancing item (“try to stay home whenever possible, even if I am not sick”), we also asked subjects a series of questions about specific activities they intended to avoid. In particular, we asked subjects to report their intentions, on 0–100 sliding scales, to avoid a set of 10 activities (“going to bars”, “going to restaurants”, “going to coffee shops”, “going to the grocery store”, “going to the gym”, “going to work (somewhere outside of your home)”, “using public transportation”, “going to the airport and flying”, “socializing in small gatherings”, and “attending large events or gatherings”). Then, on a subsequent page, we asked subjects which of these activities they would engage in at least sometimes if coronavirus were not a concern. To create a composite measure of social distancing intentions, for each subject, we averaged intentions to avoid all activities that the subject indicated they would otherwise engage in.

#### Perceived personal and public threat of coronavirus

Additionally, we measured the perceived personal threat (to the subject) and public threat (to society) of coronavirus. We measured perceived public and personal threat on separate pages in random order. Each construct was measured via two parallel questions using on 0–100 sliding scales (which we averaged to form composite variables). The questions were (i) “To what extent are you afraid for *yourself* (your community) of *contracting* (contracting and spreading) coronavirus (i.e., COVID-19)? In other words, to what extent do you fear the *personal repercussions for you* (collective repercussions for society) that would follow from *contracting* (contracting and spreading) coronavirus?” and (ii) “How large of a *personal* (societal) threat do you think coronavirus (i.e., COVID-19) poses to *you* (your community)?”, with italicized text corresponding to the personal threat measure and text in parentheses corresponding to the public threat measure. See Supplementary Sect. 6 for more detail regarding these questions, including a description of a programming error that caused a minor difference in the way that we measured personal versus public threat.

We measured these constructs as potential mediating variables, reasoning that our treatments might function by influencing the perceived (personal or public) threat of coronavirus. Yet across all of our studies, we consistently found no significant treatment effects, or differences between treatments, on either threat variable (see Supplementary Sect. 4.5 for details), suggesting that our treatments operated through other causal pathways (e.g., by increasing the perceived importance of prevention behaviors for avoiding harm to oneself or others). Thus, throughout this paper, instead of reporting analyses of our threat variables as potential mediators of our treatment effects, we instead report exploratory analyses of the associations between our threat variables and prevention intentions.

### Modifications for Study 2

Study 2 was very similar to Study 1, with a few differences. First, we recruited a target of *n* = 1200 subjects and assigned them to one of our three treatment conditions. Study 2 thus omitted the control condition that was included in Study 1 and had a larger target sample size per condition (*n* = 400 rather than *n* = 250). Second, we moved the measurement of all individual difference variables to the beginning of the study (i.e., before presenting our treatments), with the exception of performance on the Cognitive Reflection Task^37^ (which, like in Study 1, was measured last).

Third, we made a few modifications to the wording we used to measure the perceived public and personal threat of coronavirus. Specifically, for both our personal and public threat measures, we modified the first question to read “To what extent are you afraid of *contracting* (contracting and spreading) coronavirus (i.e. COVID-19) because of the consequences for *you personally* (for your community)?” Additionally, for our public threat measure, we modified the second question by replacing the phrase “societal threat” with “collective threat”.

Finally, as our dependent variable, we only measured prevention intentions (and did not include our set of questions from Study 1 about more specific social distancing intentions). We made this decision because in Study 1, our measure of prevention intentions (which also included an overall social distancing item) produced stronger evidence for treatment effects and interesting differences between treatments. In Study 2, we thus chose to focus on replicating the observed effects on our measure of prevention intentions. For this reason, our analyses also focus more extensively on prevention intentions than our set of questions about more specific social distancing intentions.

### Sample

A total of *n* = 1019 subjects started Study 1 and *n* = 1224 subjects started Study 2. To form our final samples, when we collected duplicate responses from the same IP address or Mturk worker ID, we included only the chronologically first response. We also excluded responses from subjects who did not complete all key measures (defined as our dependent measures and potential mediators). This left us with *n* = 988 subjects in Study 1 (mean age = 39, 57% male) and *n* = 1188 subjects (mean age = 37, 60% male) in Study 2.

### Analysis approach

All of our analyses use linear regression. In all models aggregating data from both Studies 1 and 2, we include a study dummy. When computing Cohen’s d values associated with regression results, we account for covariates (such as study dummies) by taking unstandardized regression coefficients (i.e., the difference between the covariate adjusted means for each group) as the numerator.

For analyses of our dependent variables, we report results (i) among all subjects, and, as a robustness check, (ii) among subjects for whom we measured our dependent variables *before* measuring our potential mediators. Although the latter analysis was not pre-registered, we include it to confirm the robustness of our results after having discovered an unexpected interaction between condition and the order in which we measured our dependent variables versus potential mediators. Specifically, in a regression predicting prevention intentions across both studies as a function of condition dummies, order, and their interactions, the interaction terms are jointly significant, *F*(3,2167) = 4.97, *p* = 0.002.

We tested three treatments and measured two dependent variables, creating six possible comparisons (both when evaluating the effectiveness of each treatment relative to the control, and when comparing pairs of treatments). Thus, in addition to reporting p-values for these comparisons, we also report q-values, which indicate the probability of making at least one false discovery across these six comparisons when rejecting the null hypothesis for any result with an equal or smaller q-value. Specifically, we report *calculated* q-values (reported as *q*_*c*_), derived from analytical calculations that conservatively assume that the tests for all six comparisons are independent from each other, and *simulated* q-values (reported as *q*_*s*_), derived from simulations of our actual data that take into account the non-independence between some tests. See Supplementary Sect. 2 for more details.

Finally, we note that in Study 1, we found some evidence that the Public treatment was relatively more effective for individuals reporting greater subjective health. Consequently, in our Study 2 pre-registration, we planned for our primary analyses to focus specifically on healthier individuals. However, evidence for an interaction between health and our Public treatment was weaker in Study 2 than in Study 1 (see Supplementary Sect. 4.6 for details). Thus, we do not feel confident focusing on health in our primary analyses, and instead report analyses of all subjects. We note, however, that analyses of healthy individuals also support our key findings from Studies 1–2.

## Results

### Comparisons of treatments to control

We begin by comparing each of our treatment conditions to the control in Study 1 (which included a control condition). We thus conduct regressions predicting each of our two DVs, taking the control condition as the baseline and including dummies for the other three conditions.

**Figure 2:**
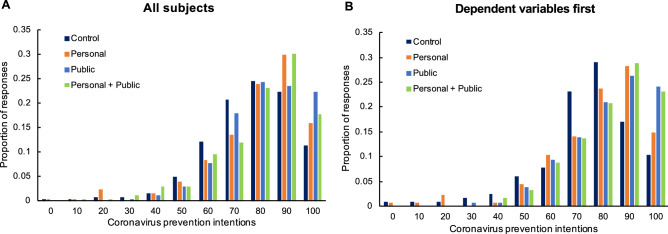
Prevention intentions by experimental condition in Study 1. Shown are frequencies of composite prevention intentions, rounded to zero or a multiple of ten, by experimental condition in Study 1, among all subjects (**A**, n = 988) and subjects for whom we measured our dependent variables first (**B**, n = 506).

When investigating composite prevention intentions (across our 11-item scale, α = 0.89 in each study), we find some robust evidence for treatment effects. We report the results of our analyses in Table 1 and plot prevention intentions across conditions in Fig. 2. We find that subjects in all treatment conditions report directionally higher prevention intentions than subjects in the control condition, but only the Public condition shows a robust treatment effect that survives corrections for multiple comparisons.

**Table 1:** Treatment effects on prevention intentions in Study 1. We compare each of our treatments to the control in Study 1. For each treatment, we report mean prevention intentions (with 95% CIs) in the treatment and control conditions, and the treatment effect (both among all subjects, and subjects for whom we measured our dependent variables first).

	All subjects (*n* = 988)	Dependent variables first (*n* = 506)
Personal	Control = 76.41 [74.31, 78.50], Personal = 79.19 [76.98, 81.40], b = 2.78, t = 1.89, d = 0.17, p = 0.059, q_c_ = 0.307, q_s_ = 0.245	Control = 74.49 [71.21, 77.77], Personal = 78.08 [74.97, 81.18], b = 3.59, t = 1.72, d = 0.21, p = 0.086, q_c_ = 0.419, q_s_ = 0.345
Public	Control = 76.41 [74.31, 78.50], Public = 81.88 [80.11, 83.64], b = 5.47, t = 3.70, d = 0.33, p < 0.001, q_c_ = 0.001, q_s_ = 0.001	Control = 74.49 [71.21, 77.77], Public = 82.39 [79.83, 84.95], b = 7.90, t = 3.74, d = 0.48, p < 0.001, q_c_ = 0.001, q_s_ = 0.001
Personal + Public	Control = 76.41 [74.31, 78.50], Personal + Public = 79.76 [77.67, 81.85], b = 3.35, t = 2.26, d = 0.20, p = 0.024, q_c_ = 0.137, q_s_ = 0.110	Control = 74.49 [71.21, 77.77], Personal + Public = 82.22 [79.69, 84.75], b = 7.73, t = 3.64, d = 0.47, p < 0.001, q_c_ = 0.002, q_s_ = 0.001

When investigating composite social distancing intentions (across our 10-item scale investigating specific social distancing intentions collected in Study 1, α = 0.91), we do not find robust evidence for treatment effects (among all subjects, all ps > 0.1 and all qs > 0.4, although effects are somewhat stronger among subjects for whom we measured our dependent variables first); see Supplementary Sect. 4.2 for more details.

### Comparisons between treatments

Next, we turn to comparing the relative effectiveness of our different treatments. When investigating prevention intentions, which we measured in both studies, to maximize precision we pool data from both studies and report analyses from this combined dataset. We note, however, that we also plot results from each study individually, to illustrate the similarity across studies. We find that the Public treatment had the directionally largest effect on prevention intentions, and thus organize our results around comparing the Public treatment to the other two treatments. Throughout this paper, we conduct this comparison via regressions that take the Public treatment condition as the baseline and measure relative effectiveness of Public using dummies for the other two treatments.

**Table 2:** Relative effects of the Public treatment on prevention intentions in Studies 1 and 2. We compare the Public treatment to each of our other treatments, across the treatment conditions of Studies 1 and 2 combined. For each comparison, we report mean prevention intentions (with 95% CIs) by condition and the relative effect of the Public treatment (both among all subjects, and subjects for whom we measured our dependent variables first).

	All subjects (*n* = 1930)	Dependent variables first (*n* = 981)
Public vs. Personal	Public = 82.48 [81.34, 83.61], Personal = 79.93 [78.66, 81.19], b = 2.55, t = 2.90, d = 0.16, p = 0.004, q_c_ = 0.022, q_s_ = 0.019	Public = 83.22 [81.69, 84.75], Personal = 79.42 [77.65, 81.19], b = 3.80, t = 3.25, d = 0.25, p = 0.001, q_c_ = 0.007, q_s_ = 0.006
Public vs. Personal + Public	Public = 82.48 [81.34, 83.61], Personal + Public = 81.07 [79.81, 82.32], b = 1.41, t = 1.60, d = 0.09, p = 0.109, q_c_ = 0.501, q_s_ = 0.408	Public = 83.22 [81.69, 84.75], Personal + Public = 83.14 [81.60, 84.67], b = 0.13, t = 0.11, d = 0.01, p = 0.913, q_c_ = 1.000, q_s_ = 1.000

**Figure 3:**
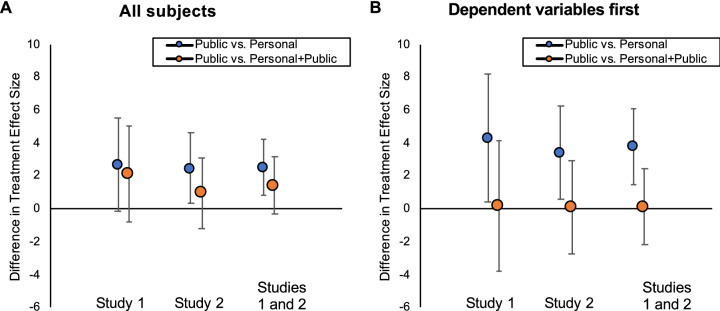
Relative effects of the Public treatment on prevention intentions in Studies 1 and 2. Shown are the relative effects of the Public treatment, as compared to the Personal treatment (blue dots) and Personal + Public treatment (orange dots). We plot results among all subjects (**A**: Study 1 n = 742, Study 2 n = 1188, Studies 1 and 2 combined n = 1930) and subjects for whom we measured our dependent variables first (**B**: Study 1 n = 389, Study 2 n = 592, Studies 1 and 2 combined n = 981).

The results, shown in Table 2 and Fig. 3, provide robust evidence that the Public treatment was more effective than the Personal treatment. (In addition to surviving corrections for multiple comparisons, this conclusion also holds when accounting for the fact that our analyses of the pooled data across studies can be conceptualized as analyses of one study in which we “peeked” at the data after an initial collection, which can inflate type-I error rate. Following the approach of Sagarin et al., 2014, we calculate that under a “worst-case scenario” approach to data peeking, an adjusted alpha threshold of 0.028 is needed to maintain an actual type-I error rate of 0.05, and the observed p and q values fall below this threshold; see Supplementary Sect. 3 for more details.) Furthermore, we find no significant difference between the effectiveness of the Public treatment and Personal + Public treatment. Thus, in Studies 1–2, we find evidence for the power of prosocial framing.

When investigating composite social distancing intentions (from our 10-item scale investigating specific social distancing intentions, which we measured only in Study 1), we find no significant differences between any pairs of our treatments in any of our analyses (all ps > 0.1 and all qs > 0.5); see Supplementary Sect. 4.2 for more details. However, the Public treatment was more effective than the Personal treatment at influencing the overall social distancing item included in our composite measure of prevention intentions (“try to stay home whenever possible, even if I am not sick”). In an aggregate analysis of Studies 1 and 2, we find a significant positive effect of a Public vs. Personal dummy on this item, both among all subjects, *b* = 3.70, *t* = 2.83, *d* = 0.16, *p* = 0.005, and subjects for whom we measured our dependent variables first, *b* = 6.11, *t* = 3.35, *d* = 0.26, *p* = 0.001. Thus, we find some evidence that the Public treatment may have been more effective than the Personal treatment at encouraging social distancing in Studies 1–2.

We also note that we find no clear evidence of heterogeneity in the effects of our treatments on prevention intentions either (i) across the 11 prevention behaviors we investigated (see Supplementary Sect. 4.3), or (ii) across individuals based on the individual difference variables we collected (see Supplementary Sect. 4.4).

Together, these results reveal that prosocial framing was more effective than self-interested framing. Thus, we find evidence that it is valuable to encourage people to consider that prevention behaviors can, in additional to conferring personal benefits, also serve to benefit others.

### Perceived public and personal threat of coronavirus

Next, we provide further support for the hypothesis that prosocial motivations play a central role in driving coronavirus prevention intentions by investigating the association between prevention intentions and the perceived public and personal threat of coronavirus.

As illustrated in Table 3, we find that (i) both threat variables were positively associated with prevention intentions, and (ii) the association with prevention intentions is significantly stronger for perceived *public* than *personal* threat (although there was substantial covariance between threat variables, *r* = 0.72 in Study 1 and *r* = 0.68 in Study 2, ps < 0.001). These results hold in both Study 1 and Study 2 (despite the fact that we measured our threat variables slightly differently across studies), and are robust to including controls in our multiple regression models for age, gender, education, income, and political party affiliation.

**Table 3:** Associations between threat variables and prevention intentions in Studies 1 and 2. We report results from regressions predicting prevention intentions as a function of our threat variables. Shown are results from (i) a set of separate regression models for each threat variable (Column 1) and (ii) multiple regression models using both threat variables (Columns 2–3), for Study 1 (top rows) and Study 2 (bottom rows). We show results from multiple regression models both with and without controls for age, gender, education (coded here and in all analyses as a college degree dummy), income, and political party affiliation. All coefficients are standardized coefficients, with standard errors for each coefficient in parentheses. For each model, we also report results from a test comparing the public versus personal threat coefficient.

	Separate models	Multiple regression	Multiple regression with controls
**Study 1 (n = 988)**			
Personal threat	0.412***	0.0730	0.0890*
	(0.0290)	(0.0393)	(0.0398)
Public threat	0.522***	0.469***	0.451***
	(0.0272)	(0.0393)	(0.0394)
Public vs. Personal comparison	t(985) = 5.40, p < 0.001	F(1,985) = 29.59, p < 0.001	F(1,980) = 24.05, p < 0.001
**Study 2 (n = 1188)**			
Personal threat	0.401***	0.0652*	0.0843**
	(0.0266)	(0.0332)	(0.0325)
Public threat	0.540***	0.496***	0.498***
	(0.0244)	(0.0332)	(0.0326)
Public vs. Personal comparison	t(1185) = 7.02, p < 0.001	F(1,1185) = 50.31, p < 0.001	F(1,1180) = 48.25, p < 0.001

Standard errors in parentheses.

***p < 0.001, **p < 0.01, *p < 0.05.

Together, these results are consistent with the proposal that prosocial motivations play an important role in driving prevention intentions.

## Study 3

We now turn to describing of our second set of studies, which were conducted during in a later stage of the COVID-19 pandemic in the United States.

## Methods

### Overview

On April 17 (Study 3a), April 22 (Study 3b), April 23 (Study 3c) and April 30 (Study 3d), 2020, we conducted four independent but almost identical “runs” of a study using convenience samples from Amazon Mechanical Turk. This set of studies (which we also refer to simply as “Study 3”) was conceptually similar to Studies 1 and 2, and again investigated the effectiveness of our three treatments (Personal, Public, and Personal + Public). However, in Study 3 we updated our stimuli to account for the progression of the pandemic, including by emphasizing the long-term threat posed by coronavirus and measuring intentions to engage in prevention behaviors even *after* the conclusion of official stay-at-home orders (which were pervasive in the U.S. in April 2020). We also made some other meaningful design changes, described below.

Across all runs of Study 3, we assigned subjects to one of our three treatments. Instead of including a no-treatment control condition, we measured prevention intentions twice: once before subjects were exposed to their treatment (at “Time 1”), and once after subjects were exposed to their treatment (at “Time 2”). This design increased our power to detect treatment effects (conceptualized in Study 3 as differences between Time 1 and Time 2 prevention intentions), and also allowed us to investigate whether the relative effectiveness of our treatments varied as a function of Time 1 prevention intentions.

We conducted four runs of Study 3 because, in each of the first three runs (but not the fourth), we observed substantial differences in Time 1 (i.e., pre-treatment) prevention intentions across conditions. While we planned, in our pre-registrations, to control for Time 1 prevention intentions when predicting Time 2 prevention intentions, we were nonetheless concerned that the Time 1 imbalances might influence our results and thus chose to continue repeating the study. However, we ultimately found qualitatively identical results when either (i) analyzing only Study 3d (in which there was no substantial Time 1 imbalance) or (ii) conducting an aggregate analysis of Studies 3a–d; for completeness, we present results from both analysis approaches.

In Studies 3a–c, we recruited *n* = 750 subjects (250 per condition). In Study 3d, (i) we recruited a larger sample of *n* = 1800 subjects (600 per condition), in order to reduce the probability of again observing imbalanced Time 1 prevention intentions across conditions, and correspondingly (ii) shortened the study by measuring fewer individual difference variables.

### Treatments

Like in Studies 1–2, our Study 3 treatments again consisted of a written text and a flier. However, as noted above, we updated our stimuli. To introduce the written text portion of our treatments, we presented subjects with the following text: “Please read the following information about COVID-19.” In all treatments, the written text then began with two paragraphs describing the ongoing threat posed by coronavirus and emphasizing the possibility of new outbreaks; see Supplementary Sect. 6 for text. The written text then preceded to a new page, which varied across treatments. The *Personal* treatment read:

For these reasons, **coronavirus is likely to remain a serious threat to you** for the foreseeable future. It is important that you continue to take this threat very seriously to prevent contracting COVID-19 and getting very ill or dying. It is recommended that you continue to take the necessary steps to **keep yourself safe** from infection now, and from new outbreaks in the future.

The *Public* treatment read:

For these reasons, **coronavirus is likely to remain a serious threat to your community** for the foreseeable future. It is important that you continue to take this threat very seriously to prevent spreading COVID-19 and causing people in your community to get very ill or die. It is recommended that you continue to take the necessary steps to **keep your community safe** from infection now, and from new outbreaks in the future.

And the *Personal* + *Public* treatment read:

For these reasons, **coronavirus is likely to remain a serious threat to you and your community** for the foreseeable future. It is important that you continue to take this threat very seriously to prevent contracting COVID-19 and getting very ill or dying, or spreading COVID-19 and causing people in your community to get very ill or die. It is recommended that you continue to take the necessary steps to **keep yourself and your community safe** from infection now, and from new outbreaks in the future.

Finally, in all treatments, the written text concluded by presenting two paragraphs encouraging subjects to continue engaging in prevention behaviors, even after official stay at home orders end, and noting the potential importance of contract tracing and testing efforts; see Supplementary Sect. 6 for text. We note that, as was true for Studies 1–2, our treatments again varied slightly in length, but the length differences were again quite minimal (especially in the context of the text that was constant across treatments).

**Figure 4:**
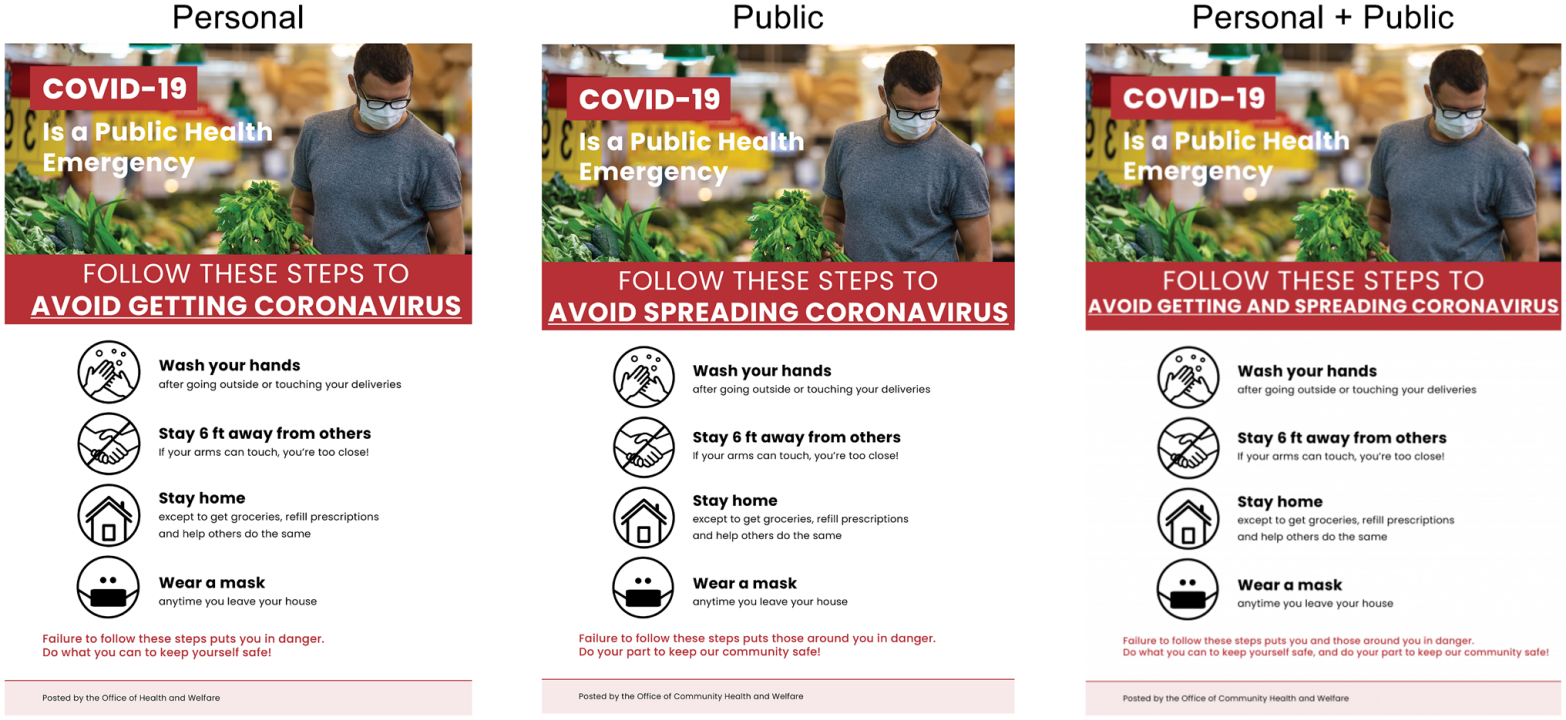
Fliers shown in each treatment across Studies 3a–d. Photograph attribution: iStock.com/Space_Cat.

We also updated our fliers, illustrated in Fig. 4. To introduce the flier portion of our treatments, in Studies 3a–c, we presented subjects with the following text: “Thank you. Now, please carefully look at this flier, which provides further guidelines for behavior over the next few weeks, while stay at home orders and other official social distancing measures remain in place”. By the time we ran Study 3d, some states were beginning to lift such measures, so we instead used the text “Thank you. Now, please carefully look at this flier, which provides guidelines for behavior relevant to COVID-19.” The images in the fliers were again purchased from the stock photo provider istockphoto.com.

### Measures

In Study 3, we began by measuring prevention intentions, and then exposed subjects to a randomly assigned treatment. Next, we re-measured prevention intentions, presented subjects with an attention check (in Studies 3a–c), measured our threat variables, and concluded by measuring a set of individual difference variables (including a second attention check in Studies 3a–c) described in Supplementary Sect. 1.

#### Prevention intentions

As in Studies 1–2, in Study 3 we measured prevention intentions via 0–100 sliding scales. To limit anchoring effects, we modified the sliders so that subjects were not shown a numeric value that corresponded to their responses. Additionally, we updated our measure of prevention intentions by (i) asking subjects specifically about their intentions “even *after* official stay-at-home orders end”, and (ii) measuring intentions to engage in a different set of 10 prevention behaviors.

Specifically, Study 3 measured intentions to “wash my hands as much as possible for the foreseeable future”, “try my hardest to avoid touching my face for the foreseeable future”, “limit my physical interaction with others when possible for the foreseeable future”, “refrain from visiting anyone who is sick at the hospital, even if they are a close family member and even if they are dying”, “if relevant, allow the government to track my health data, movements, and/or the people I interact with”, “if relevant, allow the government to regularly test me for COVID-19”, “wear a mask when I leave the house for the foreseeable future,” “completely avoid any unnecessary physical contact with others (e.g., hugging or handshakes) for the foreseeable future”, “avoid crowded indoor or outdoor spaces for the foreseeable future”, and “even if I think I have contracted and recovered from COVID-19, remain vigilant unless I have a confirmed positive test (either for the virus during infection, or antibodies after infection)”.

#### Perceived public and personal threat of coronavirus

We measured the perceived public and personal threat of coronavirus as in Study 2 (and correcting the programming error present in Studies 1–2).

### Sample

A total of *n* = 4174 subjects started Studies 3a–d and *n* = 1846 subjects started Study 3d. We formed our final Study 3 samples as in Studies 1–2, leaving us with *n* = 3985 subjects across Studies 3a–d (mean age = 38, 55% male) and *n* = 1773 subjects in Study 3d (mean age = 39, 48% male).

### Analysis approach

Because of the imbalance in Time 1 prevention intentions across treatments in Studies 3a–c, when investigating effects of our treatments on dependent measures, we report results both (i) across Studies 3a–d (and include study dummies in our models), and (ii) in Study 3d only.

As in Studies 1–2, our Study 3 analyses correct for multiple comparisons. Study 3 tested three treatments and measured one dependent variable; thus, we report q-values that indicate the probability of false discovery across three comparisons. However, because the results of Study 3 are clearly definitive (specifically, in supporting the conclusions that all treatments were effective, and there were no meaningful differences in effectiveness between treatments), we simply report (more conservative) *calculated* q-values (*q*_*c*_) and do not generate *simulated* q-values that account for the non-independent between some tests.

In our Study 3 pre-registrations, we planned to analyze our data in long format (with one observation per prevention intention item per subject). Yet for consistency with our approach from Studies 1–2, we instead primarily analyze data in wide format, and compute composite prevention intentions across our 10 items (across Studies 3a–d, Time 1 α = 0.87 and Time 2 α = 0.89). When investigating whether the relative effectiveness of treatments is influenced by Time 1 prevention intentions, however, we do analyze the data in long format (allowing us to address this research question more precisely). We note that our conclusions from all analyses are qualitatively unchanged when analyzing in wide versus long format.

## Results

### Effectiveness of treatments

We begin by investigating whether each of our treatments was effective at increasing prevention intentions. To this end, we shape our data to have two observations per subject (for composite prevention intentions at Times 1 and 2, respectively). Then, for each treatment, we predict prevention intentions as a function of time, with robust standard errors clustered on subject. As illustrated by Table 4, we find strong evidence that all three treatments were effective at increasing prevention intentions.

**Table 4:** Effectiveness of treatments in Study 3. For each treatment, we report mean Time 1 (i.e., pre-treatment) and Time 2 (i.e., post-treatment) prevention intentions, and the change over time (across Studies 3a–d, and in Study 3d only).

	Studies 3a–d (*n* = 3985)	Study 3d (*n* = 1773)
Personal	Time 1 = 70.88 [69.88, 71.89], Time 2 = 74.49 [73.48, 75.51], b = 3.61, t = 16.62, d = 0.19, p < 0.001, q_c_ < 0.001	Time 1 = 70.41 [68.87, 71.96], Time 2 = 74.01 [72.41, 75.62], b = 3.60, t = 12.36, d = 0.18, p < 0.001, q_c_ < 0.001
Public	Time 1 = 73.46 [72.50, 74.42], Time 2 = 76.73 [75.77, 77.68], b = 3.27, t = 14.99, d = 0.18, p < 0.001, q_c_ < 0.001	Time 1 = 71.23 [69.73, 72.73], Time 2 = 75.04 [73.50, 76.58], b = 3.81, t = 12.02, d = 0.20, p < 0.001, q_c_ < 0.001
Personal + Public	Time 1 = 72.07 [71.12, 73.03], Time 2 = 75.06 [74.09, 76.03], b = 2.99, t = 14.11, d = 0.17, p < 0.001, q_c_ < 0.001	Time 1 = 70.84 [69.38, 72.30], Time 2 = 74.37 [72.86, 75.89], b = 3.53, t = 10.66, d = 0.19, p < 0.001, q_c_ < 0.001

### Comparisons between treatments

Next, we turn to comparing the relative effectiveness of our different treatments. To this end, we shape our data to have one observation per subject (with two variables for composite prevention intentions at Times 1 and 2, respectively). Then, we predict Time 2 prevention intentions as a function of treatment dummies, controlling for Time 1 prevention intentions. As illustrated by Table 5, we find that our three treatments were similarly effective.

**Table 5:** Comparisons between treatments in Study 3. For each pairwise comparison, we report the relative effect of the treatment listed first on Time 2 prevention intentions, when controlling for Time 1 prevention intentions (across Studies 3a–d, and in Study 3d only).

	Studies 3a–d (*n* = 3985)	Study 3d (*n* = 1773)
Public vs. Personal	b = − 0.12, t = − 0.42, d = − 0.01, p = 0.677, q_c_ = 0.966	b = 0.25, t = 0.57, d = 0.01, p = 0.567, q_c_ = 0.919
Public vs. Personal + Public	b = 0.40, t = 1.33, d = 0.02, p = 0.182, q_c_ = 0.453	b = 0.30, t = 0.68, d = 0.02, p = 0.498, q_c_ = 0.874
Personal vs. Personal + Public	b = 0.52, t = 1.75, d = 0.03, p = 0.080, q_c_ = 0.220	b = 0.05, t = 0.10, d = 0.00, p = 0.918, q_c_ = 0.999

We also find no robust evidence that the relative effectiveness of our treatments varies as a function of Time 1 prevention intentions; see Supplementary Sect. 4.7 for more detail.

### Perceived public and personal threat of coronavirus

Next, we turn to investigating the association between prevention intentions and the perceived public and personal threat of coronavirus. Given that our threat variables did not mediate our treatment effects in Studies 1 and 2, our Study 3 pre-registrations simply planned to investigate absolute values of perceived threat (for the purpose of comparison to Studies 1 and 2). (See Supplementary Sect. 4.5 for this comparison, as well as evidence that, as in Studies 1–2, in Study 3 we again found no differences between our treatments on either threat variable).

However, we again investigate the association between prevention intentions and our threat variables in order to shed further light on the extent to which prevention intentions are motivated by prosociality versus self-interest. These analyses are not pre-registered, but nonetheless serve as a confirmatory test of whether our association results from Studies 1–2 replicate in Study 3.

As illustrated in Table 6, across Studies 3a–d we find that (i) both threat variables were associated with composite prevention intentions, and (ii) the association is significantly stronger for perceived *public* than *personal* threat (although there was substantial covariance between threat variables, *r* = 0.73). These results hold when predicting composite prevention intentions at either Time 1 or Time 2, and are robust to including controls for age, gender, education, income, political party affiliation, and race (note that we control for race in our analyses of Study 3, but not Studies 1–2, because race was only measured in Study 3).

**Table 6:** Associations between threat variables and prevention intentions in Study 3. We report results from regressions predicting prevention intentions as a function of our threat variables, across Studies 3a–d. Shown are results from (i) a set of separate regression models for each threat variable (Column 1) and (ii) multiple regression models using both threat variables (Columns 2–3), when predicting prevention intentions at Time 1 (top rows) and Time 2 (bottom rows). We show results from multiple regression models both with and without controls for age, gender, education, race, income, and political party affiliation. All coefficients are standardized coefficients, with standard errors for each coefficient in parentheses. For each model, we also report results from a test comparing the public versus personal threat coefficient.

	Separate models	Multiple regression	Multiple regression with controls
**Time 1 prevention intentions**			
Personal threat	0.505***	0.185***	0.170***
	(0.0138)	(0.0190)	(0.0191)
Public threat	0.570***	0.435***	0.437***
	(0.0131)	(0.0190)	(0.0193)
Public vs. Personal comparison	t(3982) = 6.94, p < 0.001	F(1,3979) = 49.65, p < 0.001	F(1,3967) = 55.73, p < 0.001
**Time 2 prevention intentions**			
Personal threat	0.505***	0.160***	0.145***
	(0.0138)	(0.0189)	(0.0188)
Public threat	0.587***	0.469***	0.471***
	(0.0129)	(0.0188)	(0.0190)
Public vs. Personal comparison	t(3982) = 8.70, p < 0.001	F(1,3979) = 77.43, p < 0.001	F(1,3967) = 85.30, p < 0.001

Standard errors in parentheses.

***p < 0.001, **p < 0.01, *p < 0.05.

Together, these results are consistent with the proposal that prosocial motivations play an important role in driving prevention intentions.

## Study 4

In Study 4, we investigate the generalizability of the associations between our threat variables and prevention intentions to a more representative sample and different operationalizations of perceived public and personal threat.

## Methods

To do so, we conduct a novel reanalysis of data from Pennycook et al.^38^, who used Prolific to recruit a sample of *n* = 748 American subjects on March 24th, 2020 that matched the national distribution on age, gender, ethnicity, and geographic region. Subjects completed the same prevention intentions measure from Studies 1 and 2. As in our previous analyses, we averaged responses to these items to create a composite intentions score. Additionally, subjects used 7-point Likert scales to indicate their agreement with various statements related to the threat posed by COVID-19. Our main text analysis focuses on the items that we see as mapping most closely onto the constructs of public threat (“The coronavirus poses a major threat to the public”) and personal threat (“Because of my age and/or pre-existing conditions, I am likely to have serious symptoms if I were to contract the coronavirus” and “Because of my age and/or pre-existing conditions, I am likely to need hospitalization if I were to contract the coronavirus”; averaged to create a personal threat index). In Supplementary Sect. 4.10 we demonstrate that the results are robust to alternative choices about which items to use (including using all items collected).

We note that the Study 4 correlation between perceived public threat and our perceived personal threat index (*r* = 0.24) was substantially lower than in Studies 1–3 (although still highly statistically significant, p < 0.001). This reduces any potential concerns of multiple-collinearity that may have arisen in the previous analyses.

For full methodological details and materials, see Pennycook et al.^38^ and https://osf.io/3a497/.

## Results

As illustrated in Table 7, and consistent with our results from Studies 1–3, we again find that (i) both personal and public threat are associated with prevention intentions, and (ii) the association is significantly stronger for perceived *public* than *personal* threat. Our results are also robust to including controls in the multiple regression model for age, gender, education, race, income, and political party affiliation. Thus, we find that the correlational results observed in Studies 1–3 generalize to a more representative sample, and different measures of threat.

**Table 7:** Associations between threat variables and prevention intentions in Study 4. We report results from regressions predicting prevention intentions as a function of the composition thread variables collected in Study 4. Shown are results from (i) a set of separate regression models for each threat variable (Column 1) and (ii) multiple regression models using both threat variables (Columns 2–3). We show results from multiple regression models both with and without controls for age, gender, education, race, income, and political party affiliation. All coefficients are standardized coefficients, with standard errors for each coefficient in parentheses. For each model, we also report results from a test comparing the public versus personal threat coefficient.

	Separate models	Multiple regression	Multiple regression with controls
Personal threat	0.282***	0.191***	0.179***
	(0.0351)	(0.0336)	(0.0397)
Public threat	0.418***	0.371***	0.355***
	(0.0333)	(0.0336)	(0.0354)
Public vs. Personal comparison	t(745) = 3.24, p = 0.001	F (1,745) = 11.56, p < 0.001	F (1,709) = 8.58, p = 0.004

Standard errors in parentheses.

***p < 0.001, **p < 0.01, *p < 0.05.

## Study 5

In Study 5, we provide a final source of support for the proposal that prosocial motivates contribute to prevention intentions. Specifically, we investigate the association between prevention intentions across Studies 1–3 and prosocial behavior in incentivized economic game experiments conducted years prior to the pandemic.

## Methods

To this end, we draw on an external dataset of MTurk studies conducted by members of our research group. All of these studies included an economic “Dictator Game” (DG), in which subjects received an endowment of money (which varied across studies) and were asked to allocate it between themselves and another anonymous MTurk worker (in increments that varied across studies). Subjects faced a financial incentive to selfishly keep the money for themselves: in all studies, subjects’ DG decisions influenced their bonus payment. Thus, we take the percentage of the starting endowment that subjects chose to allocate to the other Mturk worker as a measure of prosocial behavior.

This dataset compiled 44 different studies^39–41^, conducted between 2012 and 2018, and included 37,622 unique decisions made by 23,170 unique subjects (as indexed by Mturk Worker IDs). For subjects who made more than one DG decision, we computed the average across their decisions.

We then investigated the association between prevention intentions in Studies 1–3 and previous DG decisions. To this end, we merged our data from Studies 1–3 with our DG dataset (using Mturk Worker IDs), resulting in DG data for a total of *n* = 1522 subjects across Studies 1–3. To conduct an aggregate analysis of Studies 1–3, we used our composite measure of prevention intentions from Studies 1–2 and our composite measure of Time 1 prevention intentions from Studies 3a–d.

## Results

We found a small but significant positive association between prevention intentions and DG decisions (*B* = 0.08, *t* = 3.42, *p* = 0.001) that is robust to controlling for age, gender, education, race, income, and political party affiliation (*B* = 0.06, *t* = 2.54, *p* = 0.011). We note that we also find similar results when using Time 2 prevention intentions from Studies 3a–d (without controls *B* = 0.09, *t* = 3.75, *p* < 0.001, with controls *B* = 0.07, *t* = 2.69, *p* = 0.007). We also note that in our models with controls, because race was only measured in Study 3, we recoded our dummies for each racial category to include a third value for missing data.

Thus, we find evidence that prosociality, as measured by incentive-compatible behavior in the Dictator Game (conducted years prior to the coronavirus pandemic), is positively associated with coronavirus prevention intentions. This result provides further support for the proposal that prosocial motives contribute to prevention intentions.

## Study 6

Finally, in Study 6 we present a field experiment testing messaging aimed at promoting the installation of a contact tracing app. Contact tracing apps use Bluetooth on users’ mobile phones to identify which users have been in close proximity to each other. They then can notify a user if they have been in close proximity with somebody who has tested positive for COVID-19. This allows the notified individual to get tested when they otherwise might not have, and, if they test positive, to seek care earlier than they otherwise might have, and to take measures to avoid infecting others. Using such apps thus has benefits both for oneself and for others, mirroring other COVID-19 prevention behaviors. In December 2020, the time that we conducted our field experiment, contact tracing apps were available in roughly half of U.S. states and were touted by experts as one of the most effective tools in combating the spread of COVID-19 before vaccines became more widely available.

## Methods

Our field experiment was performed in collaboration with COVID Act Now (CAN). CAN is one of the three largest COVID-19 websites, as measured by daily visits. CAN provides key statistics about the pandemic at a national, state, and county level, and also sends a daily newsletter with COVID-19 news. This newsletter was the setting for our experiment.

Our experiment was performed on three consecutive days from Monday December 21 to Wednesday December 23, 2020. Our sample was the entirety of CAN’s newsletter subscribers, which numbered 152,556 at the time. Although our goal was for users to be perfectly randomized to condition, our field partner implemented the following procedure.

The sample was divided into three treatment groups. Assignment to groups was performed based on last name: subscribers with last names that began with the letters A through I were assigned to group one (*n* = 59,352), subscribers with last names that began with the letters J through Q were assigned to group two (*n* = 56,978), and the remaining subscribers were assigned to group three (*n* = 36,226).

On all 3 days of the experiment, all subscribers were emailed a newsletter. The email subject lines and content of these newsletters was different on each of these 3 days. To implement our treatments, on each day, just one of the treatment groups received a short “header” in the body of their email, at the beginning of their newsletter. This header briefly explained what contact tracing apps are and encouraged the reader to download such an app. It also provided a link where readers could see if their state had a contact tracing app available, and, if so, could download the app.

There were three versions of the header. The version sent on Monday December 21 was sent to subscribers in group one, and emphasized the *personal* benefits of downloading a contact tracing app (Personal treatment): “It lets you know if you may have COVID, which helps you obtain treatment more quickly.” The version sent on Tuesday December 22 was sent to subscribers in group two, and emphasized the benefits that downloading an app has *for others* (Public treatment): “It lets you know if you may have COVID, which prevents you from spreading COVID to more people”. Finally, the version sent on Wednesday December 23 was sent to subscribers in group three, and emphasized both benefits (Personal + Public treatment): “It lets you know if you may have COVID, which helps you obtain treatment more quickly and prevents you from spreading COVID to more people”.

For complete copies of the newsletters sent each day, see Supplementary Sect. 6.5.

## Results

Our key outcome measure is the *click-through rate*: the fraction of people who clicked the link to the contract tracing app, among those who opened the email (and thus saw the header prompting them to download the contact tracing app). As described above, our three treatments displayed the relevant header on three different dates, and in the context of three different email subject lines. Thus, overall email open rates varied slightly across days (reflecting effects either of date or email subject line); in particular, 40.28% (*n* = 23,908) opened the email on 12/21, 41.50% (*n* = 23,644) opened the email on 12/22, and 37.98% (*n* = 13,760) opened the email on 12/23. Thus, we cannot simply compare the total number of link clicks on each of these days (as differences could reflect unequal rates of opening the email, rather than the influence of our messaging in the headers). We therefore instead ask: among those who opened their email and saw their header, what fraction chose to click the link?

We find that the click-through rate in the Personal treatment (6.27%) did not differ significantly from the click-through rate in the Public treatment (6.64%); two-sample test of proportions: z = −1.64, *p* = .100. However, the click-through rate in the Personal + Public treatment (7.33%) was significantly higher than the rate observed in either the Personal treatment (z = 3.98, *p* < .001) or the Public treatment (z = 2.55, *p* = .011).

By examining actual link-clicking behavior in the field (rather than self-reported behavioral intentions in a survey experiment), Study 6 provides externally valid evidence for the power of prosocial framing. A header that highlighted the personal and public benefits of contract tracing resulted in higher click-through rates than headers highlighting either only the personal benefits or only the public benefits.

It is important to note that because of the imperfect randomization, this evidence is only suggestive. First, recall that assignment to treatment was not random, but rather based on subscriber last name. Thus, it is possible that the observed differences between treatments reflect that subscribers with different last names behave differently. Second, recall that our three treatments were presented on different dates and in the context of different email subject lines, resulting in different open rates across days. If different *types* of subscribers opened the email on different days, it is possible that the observed differences between treatments reflect that some types were more inclined to click the link than others. Finally, recall that our three treatments were presented in the context of different newsletter content (that was displayed below the header with our messaging). Thus, it is possible that the observed differences between treatments reflect that these differences in newsletter content influenced click-through rates. Thus, while Study 6 provides suggestive evidence for the power of combined personal and public framing in a field context, future work should seek to conceptually replicate these results using a more appropriate randomization procedure.

## Discussion

Coronavirus prevention efforts can reasonably be conceptualized as either self-interested or prosocial. We have investigated which framing is more effective—and motivation is stronger—for fostering intentions to engage in prevention behaviors.

First, we investigated the relative efficacy of self-interested (“don’t get it”) versus prosocial (“don’t spread it”) messaging. In a set of studies conducted earlier in the pandemic (March 14–16, 2020, at which time there were fewer than 2000 U.S. cases), prosocial framing was more effective than self-interested framing. Furthermore, combining self-interested and prosocial framing was no more effective than pure prosocial framing. In a set of studies conducted slightly later in the pandemic (April 17–30, 2020, at which time hundreds of thousands of Americans had been infected), all three framing strategies were similarly effective. Finally, in a field experiment conducted more than half a year later (December 21–23, 2020) that featured heightened external validity but imperfect randomization, we found suggestive evidence that combining self-interested and prosocial framing may have been more effective at motivating people to sign up for a contact tracing app than self-interested or prosocial framing alone.

Thus, across all of our experiments, we never found self-interested framing to be significantly more effective than prosocial framing. These findings are striking, considering the substantial risks of hospitalization and death posed by COVID-19 to individuals. And they suggest that people are receptive to the suggestion that prevention behaviors can, in additional to conferring personal benefits, also serve to benefit others. In this way, our results imply that prosocial motives—or the desire to appear prosocial—can support prevention behaviors.

We also supported this proposal with correlational analyses. Across all of our survey experiments, we consistently found that the perceived *public* threat of coronavirus is more strongly associated with prevention intentions than the perceived *personal* threat. Furthermore, we found that people who behaved prosocially in incentivized economic games conducted years before the pandemic reported greater prevention intentions. While these correlation results cannot provide decisive causal evidence for the influence of prosocial motives, they are robust to demographic controls and provide suggestive evidence that builds on our treatment effects. Together, our results thus challenge the hypotheses that self-interested motives are the dominant driver of prevention intentions, and that self-interested appeals are the best messaging strategy. And they suggest that prosociality can play an important role in helping to combat COVID-19.

Our results thus contribute to the body of research demonstrating that both self-interest and prosociality can motivate people to prevent infectious disease spread—including recent work specifically investigating efforts to prevent coronavirus^42–51^. And our findings align with evidence that people are moral actors who care for others and are motivated to avoid appearing selfish, and that regard for others is especially strong in the domain of physically aversive outcomes.

It is interesting that we found an advantage for prosocial appeals in our first set of survey experiments, but no differences in the effectiveness of our treatments in our second set of survey experiments. While our data cannot speak to the explanation for this change, one possibility is that the psychology surrounding coronavirus changed meaningfully between March and April 2020, as the pandemic progressed. Regardless, the differences between our sets of results highlight that an important goal for ongoing COVID-19 research is to continue investigating the value of prosocial framing across contexts.

Another important question pertains to the efficacy of prosocial framing across different messaging content. While our results highlight the potential power of prosocial framing, our survey experiments tested two (similar) sets of messages. When designing the treatments used in these experiments, we sought to emphasize the substantial threats posed by coronavirus to both individuals and society. However, different self-interested appeals could potentially be more effective (e.g., if they compellingly suggested that young people are at higher personal risk than most believe). Furthermore, we contrasted concern for oneself with concern for one’s community at large; concern for close others (e.g., friends and family)^49^ presents an interesting intermediate case that combines self-interested and prosocial motives differently than our Personal + Public treatment did. Thus, to gain a general understanding of the value of self-interested versus prosocial framing, it is important to investigate a variety of different messaging contexts.

Subsequent to our survey experiments, numerous other research groups have used designs that are conceptually similar to ours (i.e., that investigate the efficacy of prosocial versus self-interested framing). As these other studies were conducted at different times, on different study populations, and using different stimuli and dependent measures, they give some insight into the generalizability of our findings. Taken together, the extant body of work supports the conclusions that prosocial framings can motivate coronavirus prevention intentions, and that there is limited evidence that prosocial framings are less effective than self-interested framings.

More specifically, a few papers provide suggestive evidence that prosocial framing may be more effective than self-interested framing. Across three studies of Americans, Luttrell and Petty found that, as compared to self-focused messages, subjects perceived other-focused messages to be similarly or more persuasive^52^. They also found that other-focused messages were seen as relatively more persuasive by subjects who saw public health as a moral issue. In another study of Americans, Capraro and Barcelo measured intentions to wear a face covering across four messaging conditions (emphasizing the threat of the virus to you, your family, your community, or your country), plus a control^45^. They found that the “your community” treatment increased intentions relative to the control, while there were no other significant differences between conditions. Additionally, across one study of Turkish participants and two studies of American participants, Ceylan and Hayran found that, compared to self-interested framing, prosocially framed messages were more persuasive at encouraging social distancing^53^. And finally, in a four-wave study of Japanese participants, Sasaki et al. compared the impact of five different messages (including two other-focused, one self-focused, and one both self- and other-focused), plus a control, on measures of self-reported prevention intentions and behaviors^54^. Overall, their nuanced results suggest that the other-focused messages (and especially other-focused messages that were “gain-framed” rather than “loss-framed”) were as or more effective than the self-focused or combined messages. However, the effects of their message treatments were generally less persistent over time, and less positive (and in fact were negative for some measures), in the context of self-reported behavior than prevention intentions; and they only analyze participants who responded to all four survey waves, which has the potential to induce selection effects.

Other papers have failed to demonstrate an *advantage* for prosocial framing, but have also failed to provide clear evidence that prosocial framing is any *less* effective than self-interested framing. In a conceptual replication of our survey experiments among Japanese participants, Miyajima and Murakami evaluated messages emphasizing the benefits of prevention behaviors for you, others, you and others, or your family; they found that all of these messages increased prevention intentions relative to a control, and that there were no efficacy differences between messaging strategies^55^. In a study of Danish participants, Falco and Zaccagni measured the impact of four text-message reminders treatments (emphasizing the threat to you, your family, others, or the country), and a control, on intentions to stay at home—and, in a follow-up survey, reports of having stayed at home^56^. They found that only the “you” and “family” treatments increased intentions to stay home, relative to the control, and none of their treatments had a significantly positive effect on self-reported behavior in the follow-up; however, their follow-up survey showed high attrition rates, which has the potential to induce selection effects and undermine causal inference. In a study of French participants, Hacquin et al. manipulated the language of two public health posters across a variety of treatment conditions (including one that used other-focused language and one that used self-focused language) and compared each treatment to a control poster^57^. They found no significant difference in prevention behavior intentions across any of their conditions. And finally, in a study of Americans, Favero and Pedersen compared four messaging treatments (including one self-focused and three other-focused) to a control and found no significant differences between any of their conditions in intentions to engage in social distancing^58^.

Finally, one study did find some evidence for a relative *disadvantage* of prosocial framing. In a field experiment of American Facebook users, Banker and Park evaluated the efficacy of advertisements linking users to recommendations from the CDC website^59^. They found that messages using a “distant” prosocial frame (“protect your community”) resulted in lower click-through rates than messages using a self-interested frame (“protect yourself”). However, messages using a “close” prosocial frame (“protect your loved ones”) were as effective as self-interested messages.

Thus, a variety of studies have now compared the relative efficacy of prosocial versus self-interested framing across a range of messaging contexts. Several of these studies have provided suggestive evidence for an advantage of prosocial framing, while several others have not found clear differences between the efficacy of these framings. Importantly, one study did find evidence that “distant” prosocial framing was less effective than self-interested framing, although it also found that “close” prosocial framing was no less effective. Together, this body of work is broadly consistent with the conclusions that prosocial framings can be effective, and that there is limited evidence for contexts in which they are less effective than self-interested framings.

Our work has important limitations. First, as is common in research investigating disease prevention behavior, we mostly focused on self-reported intentions for prevention behaviors (which may be susceptible to socially desirable responding). It is thus possible that prosocially-framed messaging may not be effective for changing actual behavior. Indeed, the aforementioned studies^54,56^ that measured both intentions (immediately after treatment) and self-reported behavior (at follow-up) highlight that messaging interventions may sometimes be more effective at changing intentions than behavior.

However, it is difficult to draw decisive conclusions from these studies, given that (i) attrition (from the initial survey to the follow-up) can challenge causal inference and (ii) retrospective self-reporting of behavior may not always reliably index actual behavior. Furthermore, despite its imperfect randomization, the field experiment we present in Study 6 provides suggestive evidence that adding prosocial framing to self-interested framing may motivate actual behavior. And abstracting away from COVID-19 specifically, meta-analytic evidence suggests that health interventions that induce changes in intentions do typically translate into changes in behavior^60^.

Moreover, even if social pressure to report prevention intentions did contribute to the effectiveness of prosocial messaging, our results may still be relevant. If prosocial messaging heightens social pressure to report prevention intentions, it may also heighten social pressure for actual behavior. And a great deal of research shows the power of social pressure for promoting cooperation outside the laboratory^61^. Nonetheless, it is critical that future work continue to investigate the impact of prosocially-framed messaging on actual prevention behavior.

Another important limitation of our studies is that we primarily used convenience samples of Americans recruited from Mturk, leaving open questions about the generalizability of our results. In particular, Mturk samples tend to skew younger than the national age distribution, limiting our ability to draw inferences about older people—who are a very important and vulnerable population in the context of COVID-19. That said, we do believe that understanding the motives of young people is also essential: young people tend to be less compliant with prevention behaviors, and thus are a critical target for COVID-19 messaging. Yet future work should nonetheless evaluate prosocially-framed messaging both among representative samples of Americans, and samples of vulnerable populations. In addition to older adults, evidence suggests that people from racial and ethnic minority groups are being disproportionately affected by COVID-19^62^, making them another important study population.

Relatedly, future work should continue to investigate the power of prosocial appeals across cultures. It is interesting that our results highlight the power of prosocial motives, given that the United States is a fairly individualist (rather than collectivist) culture^63^. And it is also interesting that the aforementioned set of related studies serves to broadly support our conclusions, despite sampling subjects from a variety of different countries. Further research should continue to evaluate the efficacy of prosocial framing in different cultural contexts.

## Supplementary Information

Supplementary Information.

## Data Availability

The datasets generated during and analysed during the current study are publicly available in the on OSF at https://osf.io/sr4n9/.
