# Supplementary material for: Don’t get it or don’t spread it: comparing self-interested versus prosocial motivations for COVID-19 prevention behaviors
Source: Sci Rep. 2021 Oct 12;11:20222. doi: 10.1038/s41598-021-97617-5 (PMC8511002; doi:10.1038/s41598-021-97617-5)
Supplement: Supplementary file 1 — Supplementary Information. [file 41598_2021_97617_MOESM1_ESM.pdf]

Supplementary information
for
Don't get it or don't spread it: Comparing self-interested versus prosocial motivations for COVID-19 prevention behaviors

Jillian J. Jordan, Erez Yoeli, and David G. Rand

1. Individual difference measures collected in Studies 1-3 2
2. Procedure for calculating q-values 2
  2.1 Calculated q-values 2
  2.2 Simulated q-values 3
3. Procedure for accounting for data "peeking" in Studies 1-2 4
4. Supplementary analyses 4
  4.1 Association between prevention intentions and age across Studies 1-4 4
  4.2 Analyses of social distancing intentions in Study 1 5
  4.3 Heterogeneity of treatment effects across prevention behaviors in Studies 1-2 6
  4.4 Heterogeneity of treatment effects across individuals in Studies 1-2 8
  4.5 Perceived public and personal threat of coronavirus across conditions in Studies 1-3 9
  4.6 Moderation by subjective health in Studies 1-2 10
  4.7 Did the relative effectiveness of treatments depend on Time 1 prevention intentions in Study 3? 11
  4.8 Analyses of "English check" in Studies 1-2 and attention checks in Study 3 12
  4.9 Alternative choices about public and personal threat items in Study 4 13
5. Discussion of pre-registered analysis plans 16
  5.1 Studies 1-2 16
  5.2 Study 3 17
6. Experimental materials 18
  6.1 Written text from treatments 18
  6.2 Dependent variables 20
  6.3 Perceived public and personal threat of coronavirus 24
  6.4 Individual difference variables 26
  6.5 Field experiment materials 30

1. Individual difference measures collected in Studies 1-3

As mentioned in the main text, in Studies 1-3 we collected a set of individual difference variables. Here, we describe these variables; for exact wording for all questions, see SOM Section 6.

In Studies 1-2, we measured, in a fixed order, age, gender, level of education, zip code, subjective health, number of pre-existing health conditions (from a list of conditions we specified), income bracket, political ideology (via three questions about political party identification, position on social issues, and position on fiscal issues), and previous exposure to information about COVID-19. Next, we presented subjects with a three-item Cognitive Reflection Task. Finally, we asked subjects to answer a simple analogy question and write a few sentences about their plans for the day; these measures were designed to screen for subjects who did not speak English (see SOM Section 4.7 for analyses).

In Studies 3a-c, we measured the same set of individual differences variables as in Studies 1-2, with the exceptions that that we (i) added measures of race and previous participation in surveys about COVID-19, (ii) only included one measure of political ideology (political party identification), and (iii) replaced our "English check" questions with two attention check questions (in which the question text instructed attentive subjects to select specific answer choices; see SOM Section 4.7 for analyses).

Study 3d was identical to Studies 3a-c, with the exception that, for brevity, we omitted our measures of pre-existing health conditions, zip code, and previous exposure to information about COVID-19, as well as both attention checks and the cognitive reflection task.

2. Procedure for calculating q-values

As discussed in the main text, in Studies 1-2 we tested three treatments and measured two dependent variables (DVs), creating six possible comparisons, and in Study 3 we tested three treatments and measured one DV, creating three possible comparisons. Thus, in addition to reporting p-values for these comparisons, we also report q-values, which indicate the probability of making at least one false discovery across the set of comparisons when rejecting the null hypothesis for any result with an equal or smaller q-value. We note that in Studies 1-2, we do not account for our analyses of subjects for whom we measured our dependent variables first as a separate set of comparisons, because we simply include these analyses as a robustness check (and not an independent opportunity to support a given hypothesis).

For both sets of studies, we report *calculated* q-values (reported as q_c), derived from analytical calculations that conservatively assume that the tests for all six comparisons are independent from each other. And for Studies 1-2 only, we also report *simulated* q-values (reported as q_s), derived from simulations of our actual data that take into account the non-independence between some tests. (As noted in the main text, we did not generate simulated q-values for Study 3 because the results are clearly definitive in supporting the conclusions that all treatments were effective, and there were no meaningful differences in effectiveness between treatments.)

Here we provide more details about how we derived these q values; full code to reproduce our simulations is available here.

2.1 Calculated q-values

To calculate q-values analytically, we compute q as 1-(1-p)^n, where n is the number of comparisons (i.e., six in Studies 1-2 and three in Study 3). Our calculated q-values thus represent

the probability of making at least one false discovery across the full set of tests at the relevant p-value threshold, assuming that all tests are independent from each other.

2.2 Simulated q-values

For Studies 1-2, we also compute simulated q-values, which account for the fact that the assumption of independence is overly conservative, given that (i) our two dependent variables are correlated in Study 1 ($r = .59$, $p < .001$), and (ii) the three pairwise comparisons are not independent.

Each of our simulations estimate the expected probability of making at least one false discovery across 10,000 simulation rounds. In each round, we (i) randomly sample observations (with replacement) from our data, (ii) assign each observation to a random condition (thus forcing the null hypothesis to be true), and then (iii) conduct the six relevant tests. We then calculate simulated q-values for a given p-value as the proportion of simulation rounds in which the minimum simulated p-value across the six tests is smaller than the given p-value. Below, we provide more detail about our simulations; note that we refer to prevention intentions (which we measured in both studies) as DV1, and social distancing intentions (which we measured only in Study 1) as DV2.

First, we describe the process we use to simulate q-values for the reported comparisons of our treatments to the control condition in Study 1, for both DV1 (main text Table 1) and DV2 (SOM Table S1). In each simulation round, we (i) sample from all conditions of our Study 1 data, with *n* equal to the number of observations across all conditions of Study 1, and then (ii) conduct six tests by comparing each treatment to the control for each DV.

Next, we describe the process we use to simulate q-values for the reported pairwise comparisons between our treatments. To conduct pairwise comparisons between treatments for DV1 (main text Table 2), we analyzed pooled data from Studies 1 and 2 (because both studies measured DV1). In contrast, to conduct pairwise comparisons between treatments for DV2 (SOM Table S2), we analyzed data from Study 1 only (because only Study 1 measured DV2). Accordingly, we conduct two distinct simulations to generate q-values for our analyses of DV1 and DV2, respectively. However, because there are six total comparisons across our two DVs, we use each of these simulations to determine the probability of making at least one false discovery across six tests; thus, each simulation samples data from *both* DVs.

Therefore, to generate q-values for our pairwise comparisons for DV2, in each simulation round, we (i) sample from the treatment conditions of our Study 1 data, with *n* equal to the number of observations across the treatment conditions of Study 1, and then (ii) conduct six tests by comparing each pair of treatments for each DV.

In contrast, to generate q-values for our pairwise comparisons for DV1, in each simulation round, we (i) sample from the treatment conditions of our Study 1 data, with *n* equal to the number of observations across the treatment conditions of Studies 1 and 2, and then (iii) conduct six tests by comparing each pair of treatments for each DV. We note that we sample exclusively from the Study 1 data (despite actually having analyzed data from both studies) because we did not collect DV2 in Study 2; thus, only our Study 1 data allow us to account for the correlation between DV1 and DV2 when simulating the probability of false discovery across the six tests. While the distributions of DV1 values are similar in Studies 1 vs. 2, mean values of DV1 are slightly higher in Study 2, so this approach is only approximate. We also note that to simulate the precise approach we used in our analyses, our simulations categorize observations

as belonging to "Study 1" or "Study 2" (with the sample sizes corresponding to the actual sizes of Study 1 and Study 2), and each test includes a "study" dummy in the model.

Finally, we note that when we report simulated q-values for subpopulations in our data (specifically, subjects for whom we measured our dependent variables first, in the main text, and subjects reporting above-median subjective health, in SOM Section 4.5), they come from independent simulations that sample exclusively from those subpopulations (and sample proportionately fewer observations).

3. Procedure for accounting for data "peeking" in Studies 1-2

In the main text, we show that the Public treatment was more effective than the Personal treatment at increasing prevention intentions via an analysis of the pooled data from Studies 1 and 2. As noted in the main text, our analyses of the pooled data across studies can be conceptualized as analyses of one study in which we "peeked" at the data after an initial collection, which can inflate type-I error rate. However, our conclusion that the Public treatment was more effective than the Personal treatment is robust to accounting for peeking.

Using Sagarin, Ambler, and Lee's (2014) method to evaluate augmented datasets that are based on peeking at marginally significant results, we calculated that, to maintain an actual type-I error rate of .05, it is necessary to evaluate statistical significance in our pooled dataset using an alpha threshold ranging from a "best-case scenario" of .0471 to a "worst-case scenario" of .0281 (for our analysis of all subjects; for our robustness check analysis of subjects for whom we measured our dependent variables first, the range is .049999 to .0283). We report a range, because the required alpha threshold depends on the maximum p-value observed in Study 1 for which we would have conducted Study 2 rather than declaring the initial results non-significant; this could range from a "best-case scenario" of the p-value observed in Study 1 (.066 among all subjects and .029 among subjects for whom we measured our DVs first) to a "worst-case scenario" of 1. Looking to the Public vs. Personal comparison in the main text Table 2, we note that all p- and q-values are below these "worst-case scenario" thresholds, reflecting that our results are still significantly significant after accounting for peeking.

4. Supplementary analyses

4.1 Association between prevention intentions and age across Studies 1-4

As noted in the main text, Mturk samples tend to skew young (as compared to the U.S. national age distribution). Although not representative, this skew may make Mturk samples valuable for evaluating the effectiveness of messaging, given that young people are less likely to engage in prevention behaviors. Here, we support this claim via an analysis of the association between age and prevention intentions.

First, we report an aggregate analysis of our Mturk studies (i.e., Studies 1-3, total n = 6,161). To conduct this analysis, we used our composite measure of prevention intentions from Studies 1-2 and our composite measure of Time 1 prevention intentions from Studies 3a-d. We found a significant positive association between prevention intentions and age (without controls $B = .10$, $t = 7.93$, $p < .001$, in a model with controls for gender, education, race, income, and political party affiliation $B = .10$, $t = 8.04$, $p < .001$). We note that we also find similar results when using Time 2 prevention intentions from Studies 3a-d (without controls $B = .11$, $t = 8.67$, $p < .001$, with demographic controls $B = .11$, $t = 8.61$, $p < .001$). We also note that in our models

with controls, because race was only measured in Study 3, we recoded our dummies for each racial category to include a third value for missing data.

Finally, we report an analysis of our more representative Prolific study (i.e., Study 4, *n* = 748). In this sample, we likewise found a significant positive association between prevention intentions and age (without controls *B* = .13, *t* = 3.52, p < 0.001, with demographic controls *B* = .15, *t* = 4.05, p < 0.001).

4.2 Analyses of social distancing intentions in Study 1

As reported in the main text, we do not find robust evidence of treatment effects, or differences between treatments, on our measure of composite social distancing intentions collected in Study 1. In Table S1, we report the effects of each of our treatments (relative to the control condition), and in Table S2, we report pairwise comparisons between each treatment pair.

	**All subjects (*n* = 985)**	**Dependant variables first (*n* = 505)**
**Personal**	Control = 66.50 [63.69, 69.32],<br>Personal = 69.56 [66.87, 72.25],<br>**b = 3.06, t = 1.58, d = 0.14**,<br>p = .114, q_c = .518, q_s = .419	Control = 62.91 [58.74, 67.07],<br>Personal = 68.10 [64.34, 71.85],<br>**b = 5.19, t = 1.92, d = 0.24**,<br>p = .056, q_c = .292, q_s = .238
**Public**	Control = 66.50 [63.69, 69.32],<br>Public = 69.47 [66.87, 72.08],<br>**b = 2.97, t = 1.53, d = 0.14**,<br>p = .127, q_c = .557, q_s = .456	Control = 62.91 [58.74, 67.07],<br>Public = 68.59 [64.85, 72.32],<br>**b = 5.68, t = 2.07, d = 0.26**,<br>p = .039, q_c = .210, q_s = .172
**Personal + Public**	Control = 66.50 [63.69, 69.32],<br>Personal+Public = 69.60 [66.93, 72.27],<br>**b = 3.10, t = 1.58, d = 0.14**,<br>p = .114, q_c = .516, q_s = .416	Control = 62.91 [58.74, 67.07],<br>Personal+Public = 71.83 [68.54, 75.11],<br>**b = 8.92, t = 3.23, d = 0.42**,<br>p = .001, q_c = .008, q_s = .006

Table S1. Treatment effects on social distancing intentions. *We compare each of our treatments to the control in Study 1. For each treatment, we report mean prevention intentions (with 95% CIs) in the treatment and control conditions, and the treatment effect (both among all subjects, and subjects for whom we measured our dependent variables first).*

	All subjects (*n* = 739)	Dependant variables first (*n* = 388)
**Public vs. Personal**	Public = 69.47 [66.87, 72.08],<br>Personal = 69.56 [66.87, 72.25],<br>**b = -0.09, t = -0.05, d = -0.00,**<br>p = .963, q_c = 1.000, q_s = 1.000	Public = 68.59 [64.85, 72.32],<br>Personal = 68.10 [64.34, 71.85],<br>**b = 0.49, t = 0.19, d = 0.02**,<br>p = .851, q_c = 1.000, q_s = 1.000
**Public vs. Personal+Public**	Public = 69.47 [66.87, 72.08],<br>Personal+Public = 69.60 [66.93, 72.27],<br>**b = -0.13, t = -0.07, d = -0.01,**<br>p = .948, q_c = 1.000, q_s = 1.000	Public = 68.59 [64.85, 72.32],<br>Personal+Public = 71.83 [68.54, 75.11],<br>**b = -3.24, t = -1.23, d = -0.15**,<br>p = .220, q_c = .774, q_s = .638
**Pesonal vs. Personal+Public**	Personal = 69.56 [66.87, 72.25],<br>Personal+Public = 69.60 [66.93, 72.27],<br>**b = -0.04, t = -0.02, d = -0.00,**<br>p = .985, q_c = 1.000, q_s = 1.000	Personal = 68.10 [64.34, 71.85],<br>Personal+Public = 71.83 [68.54, 75.11],<br>**b = -3.73, t = -1.43, d = -0.18**,<br>p = .154, q_c = .632, q_s = .503

Table S2. Comparisons between treatments on social distancing intentions. *We conduct pairwise comparisons across the treatment conditions of Study 1 (both among all subjects, and subjects for whom we measured our dependent variables first).*

4.3 Heterogeneity of treatment effects across prevention behaviors in Studies 1-2

Here, we investigate whether there is meaningful heterogeneity of treatment effects across prevention behaviors in Studies 1-2. As discussed in the main text, our primary analyses of prevention intentions investigated composite prevention intentions, computed by averaging intentions to engage in our set of eleven individual prevention behaviors. Here, we consider this set of behaviors individually.

In Figure S1A, we plot overall treatment effects (i.e., effects of a “treatment vs. control” dummy) on each individual prevention behavior in Study 1. In Figure S1B, we plot effects of the Public treatment, relative to the other two treatments, on each individual prevention behavior across Studies 1 and 2. We show results both among all subjects, and subjects for whom we measured our dependent variables first.

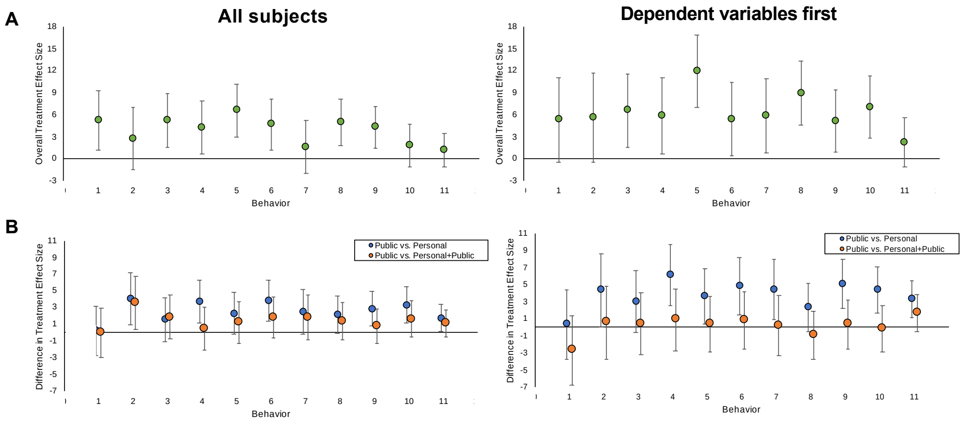

Behavior legend

1: Stock up on cleaning supplies (Baseline = 64.94)
2: Wash hands 10x a day (Baseline = 68.22)
3: Purchase food reserves and meds (Baseline = 70.87)
4: Stay home whenever possible (Baseline = 73.85)
5: Stop hugging (Baseline = 75.20)
6: Try hardest to avoid touching face (Baseline = 75.43)
7: Wash hands as much as possible (Baseline = 75.94)
8: Stop shaking hands (Baseline = 81.07)
9: Stay home if even a little bit sick (Baseline = 81.69)
10: Wash hands more often (Baseline = 83.89)
11: Cover cough and sneeze (Baseline = 89.39)

Figure S1. Treatment effects on individual prevention behaviors. *(A) Overall treatment effects on individual behaviors. Shown are the aggregated effects of our three treatments, as compared to the control condition (green dots), in Study 1 (among all subjects, n = 988, and subjects for whom we measured our dependent variables first, n = 506). (B) Effects of the Public treatment on individual behaviors. Shown are the relative effects of the Public treatment, as compared to the Personal treatment (blue dots) and Personal+Public treatment (orange dots), across the treatment conditions of Studies 1 and 2 combined (among all subjects, n = 1930, and subjects for whom we measured our dependent variables first, n = 981). Behaviors are organized in ascending order by "baseline" intentions to engage in the behavior (defined by mean intentions in the control condition of Study 1, among all subjects), and this value is also reported in the behavior legend. Error bars are 95% CIs.*

Figure S1 reveals that our overall treatment effect, and the advantage of our Public treatment relative to other treatments, are relatively robust across individual prevention behaviors. Confirming this visual impression, we find no significant heterogeneity across individual prevention behaviors. To test for heterogeneity for each condition contrast in Figure S1, we reshaped our data to long format (with one observation for each prevention intention item). We then performed a joint significance test on the interaction terms between a dummy for the relevant condition contrast, and dummies for each of the prevention intention items (with robust standard errors clustered on subject).

In analyses of all subjects, we found no significant heterogeneity across behaviors for (i) the contrast between treatments and control in Study 1, $F(10,987) = 1.38$, $p = .183$, or (ii) the contrasts between Public and Personal, $F(10,1929) = 1.51$, $p = .131$, or Public and Personal+Public, $F(10,1929) = 0.91$, $p = .519$, across Studies 1 and 2. Likewise, in analyses of all subjects for whom we measured our dependent variables first, we found no significant heterogeneity across behaviors for (i) the contrast between treatments and control in Study 1, $F(10,505) = 1.65$, $p = .090$, or (ii) the contrasts between Public and Personal, $F(10,980) = 1.54$, $p = .121$, or Public and Personal+Public, $F(10,980) = 1.31$, $p = .218$, across Studies 1 and 2.

Thus, we find no evidence of heterogeneity in treatment effects across individual behaviors.

4.4 Heterogeneity of treatment effects across individuals in Studies 1-2

Next, we investigate potential heterogeneity of treatment effects across individuals in Studies 1-2. Specifically, in Table S3, we report a set of exploratory analyses investigating whether each of our individual difference variables moderate our treatment effects.

	**All subjects**			**Dependant variables first**		
	All treatments vs. Control in Study 1	Public vs. Personal in Studies 1-2	Public vs. Pesonal + Public in Studies 1-2	All treatments vs. Control in Study 1	Public vs. Personal in Studies 1-2	Public vs. Pesonal + Public in Studies 1-2
**Age**	**0.170**	**0.120**	**0.0688**	**0.228**	**0.169**	**-0.0218**
	(0.118)	(0.0903)	(0.0906)	(0.177)	(0.127)	(0.129)
**Male**	**-0.0365**	**-0.0352**	**-0.00150**	**-0.130**	**0.00771**	**-0.0259**
	(0.0717)	(0.0440)	(0.0444)	(0.102)	(0.0641)	(0.0624)
**College degree**	**0.0848**	**0.0410**	**0.0248**	**0.155**	**0.0352**	**0.0560**
	(0.0762)	(0.0498)	(0.0477)	(0.109)	(0.0710)	(0.0668)
**Subjective health**	**-0.0922**	**0.251**	**0.326***	**-0.0225**	**0.102**	**0.200**
	(0.169)	(0.141)	(0.135)	(0.221)	(0.198)	(0.187)
**Pre-existing health conditions**	**-0.0339**	**-0.0496**	**-0.0455**	**-0.0555**	**-0.0399**	**0.00773**
	(0.0644)	(0.0375)	(0.0366)	(0.0939)	(0.0591)	(0.0546)
**Income**	**0.166**	**0.142**	**0.185***	**0.251**	**0.123**	**0.179**
	(0.105)	(0.0746)	(0.0733)	(0.147)	(0.106)	(0.103)
**Conservative (vs. liberal) political ideology: Party identification**	**-0.0292**	**-0.0634**	**-0.128***	**-0.00122**	**-0.146**	**-0.143**
	(0.0867)	(0.0606)	(0.0594)	(0.120)	(0.0895)	(0.0842)
**Conservative (vs. liberal) political ideology: Social issues**	**-0.0532**	**-0.0451**	**-0.0198**	**-0.0194**	**-0.133**	**-0.0616**
	(0.0864)	(0.0579)	(0.0569)	(0.120)	(0.0835)	(0.0802)
**Conservative (vs. liberal) political ideology: Fiscal issues**	**-0.0512**	**0.0334**	**-0.00500**	**0.0888**	**-0.0377**	**-0.0475**
	(0.0910)	(0.0623)	(0.0616)	(0.128)	(0.0898)	(0.0872)
**Previous exposure to COVID info**	**-0.120**	**0.0649**	**-0.116**	**-0.0632**	**-0.210**	**-0.237**
	(0.139)	(0.0933)	(0.0953)	(0.188)	(0.130)	(0.136)
**CRT score**	**0.000501**	**0.0154**	**-0.0149**	**0.0834**	**-0.0305**	**-0.0672**
	(0.0747)	(0.0456)	(0.0459)	(0.108)	(0.0638)	(0.0642)
**Log-transformed population density (from zip)**	**0.136**	**-0.0390**	**-0.0325**	**0.135**	**-0.174**	**-0.0741**
	(0.133)	(0.107)	(0.109)	(0.177)	(0.154)	(0.156)

Standard errors in parentheses
*** p<0.001, ** p<0.01, * p<0.05

Table S3. Individual difference variables as moderators of treatment effects. *Here we explore the extent to which our individual difference variables moderate our treatment effects. We report results from regressions predicting prevention intentions as a function of our individual difference variables, relevant condition contrasts, and their interactions, among all subjects (Columns 1-3) and subjects for whom we measured our dependent variables first (Columns 4-6). For each individual difference variable (in a series of separate regression models), shown is the interaction with (i) the overall treatment effect relative to control in Study 1 (Columns 1 and 4), and (ii) effects of the Public treatment relative to each of our other treatments, across the treatment conditions of Studies 1 and 2 (Columns 2-3 and 5-6). All coefficients are standardized coefficients, and standard errors are reported below each coefficient in parentheses. Before conducting these analyses, we (i) computed a "college degree" dummy from our measure of education, (ii) computed CRT scores (as the number of questions correct out of a possible three),*

and (iii) natural log-transformed our measure of population density. For our analyses of the overall treatment effect in Study 1, among all subjects n = 988 for all variables except population density, for which n = 954, and among subjects for whom we measured our dependent variables first, n = 506 for all variables except population density, for which n = 487. For our analyses of the Public treatment effects across Studies 1 and 2, among all subjects n = 1930 for all variables except population density, for which n = 1845, and among subjects for whom we measured our dependent variables first, n = 981 for all variables except population density, for which n = 935.

Table S3 reveals that we find no compelling evidence for moderation of our treatment effects. We find no significant moderation in our analyses of subjects for whom we measured our dependent variables first. In our analyses of all subjects, we also find no significant moderation of the overall treatment effect or the comparison of the Public vs. Personal treatments.

We do, however, find three significant moderators of the comparison of the Public vs. Personal+Public treatments. Specifically, as compared to Personal+Public, we observe relatively larger effects of the Public treatment among individuals who report higher subjective health, higher income, and stronger identification with the Democratic party. However, we note that for two of the significant interactions, conceptually related variables showed null effects (specifically, subjective health is conceptually related to pre-existing health conditions, and identification with the Democratic party is related to our other two political ideology variables). Furthermore, because our moderation analyses are only exploratory and we do not take them as strong evidence of any claims, Table S3 does not report q-values; however, it of course reports many tests, creating a multiple comparisons problem.

Thus, we ultimately do not see Table S3 as providing compelling evidence for moderation (without replication).

4.5 Perceived public and personal threat of coronavirus across conditions in Studies 1-3

Next, we discuss the perceived public and personal threat of coronavirus across conditions in Studies 1-3. We note that we analyze Studies 1 and 2 separately, because we measured these threat variables via slightly different wording in Studies 1 vs. 2; Studies 3a-d used the Study 2 wording. We also note that in Studies 1-2, due to a programming error, our personal vs. public threat variables differed in the order in which the two questions for each construct were presented; this error was corrected in Studies 3a-d. See "Experimental materials" for more detail on the measurement of threat variables across studies.

As noted in the main text, across both Studies 1-2 and Studies 3a-d, neither of our threat variables differed significantly across conditions. In Table S4, we report descriptive statistics for each threat variable across conditions, both for our earlier studies (with separate results for Study 1 and Study 2) and later studies (with separate results for Studies 3a-d and Study 3d). Table S4 also allows interested readers to compare perceived threat levels earlier vs. later in the pandemic.

	Earlier studies			
	**Study 1 (*n* = 988)**		**Study 2 (*n* = 1188)**	
	**Personal threat**	**Public threat**	**Personal threat**	**Public threat**
**Control**	54.54 [50.79, 58.29]	67.87 [64.79, 70.95]		
**Personal Treatment**	58.82 [55.21, 62.43]	70.57 [67.55, 73.59]	57.9 [55.14, 60.66]	70.4 [68.2, 72.6]
**Public Treatment**	54.75 [51.19, 58.31]	71.1 [68.17, 74.02]	57.15 [54.24, 60.05]	69.56 [67.25, 71.86]
**Personal + Public Treatment**	55.65 [51.91, 59.39]	71.31 [68.52, 74.11]	57.57 [54.65, 60.49]	70.44 [68.23, 72.66]
	**Later studies**			
	**Studies 3a-d (*n* = 3985)**		**Study 3d (*n* = 1773)**	
	**Personal threat**	**Public threat**	**Personal threat**	**Public threat**
**Personal Treatment**	60.59 [59.07, 62.12]	68.99 [67.71, 70.27]	57.21 [54.85, 59.58]	66.39 [64.36, 68.43]
**Public Treatment**	60.93 [59.39, 62.47]	70.58 [69.33, 71.83]	56.67 [54.28, 59.06]	67.11 [65.07, 69.15]
**Personal + Public Treatment**	60.5 [59.02, 61.99]	69.23 [67.98, 70.47]	57.17 [54.95, 59.39]	67.26 [65.34, 69.19]

Table S4. Threat variables by study and condition. *We report mean values (with 95% CIs) for each threat variable by study and condition.*

In statistical analyses, we find no evidence that our threat variables differed across conditions. First, looking to Studies 1-2, for each threat variable we (i) compared each treatment to the control in Study 1, and (ii) compared each pair of treatments both within Study 1 and within Study 2. Next, looking to Study 3, for each threat variable we compared each pair of treatments, controlling for composite Time 1 prevention intentions, both within Studies 3a-d and within Study 3d. We found no significant results (all ps > .05), suggesting that our threat variables do not explain our treatment effects; thus, we do not report mediation analyses.

4.6 Moderation by subjective health in Studies 1-2

Next, we discuss moderation by subjective health across Studies 1-2. As noted in the main text, in Study 1, we found some evidence that individuals reporting greater subjective health showed relatively larger effects of the Public treatment on prevention intentions. This result makes theoretical sense: healthy individuals are at lower risk for coronavirus, and thus should be less likely to see prevention behaviors as self-interested and more likely to treat them like a public good. Thus, in our Study 2 pre-registration, we planned for our primary analyses to focus specifically on healthier individuals (defined as individuals reporting subjective health above the Study 1 median). However, evidence for an interaction between health and our Public treatment effects was weaker in Study 2 than in Study 1. Thus, despite the fact that moderation by subjective health makes theoretical sense, we did not feel confident focusing on health in our primary analyses, and instead chose to report main effects among all subjects. And our pre-registrations for and analyses of Study 3 also focus on main effects among all subjects

In Table S5, however, we report detailed analyses of subjective health in Studies 1-2. Our objective in doing so is to provide transparency with respect to our pre-registered plan to focus on health in Study 2. Thus, because our pre-registrations only planned analyses of all subjects, for brevity in this section we do not additionally report analyses of subjects for whom we measured our dependent variables first.

Specifically, Table S5 reports the effects of the Public treatment on prevention intentions, relative to our other treatments, as a function of subjective health. We conduct separate analyses of the treatment conditions of Study 1 (Column 1), Study 2 (Column 2), and Studies 1 and 2 combined (Column 3). In each analysis, we compare the Public treatment to the Personal treatment (top rows), and to the Personal+Public treatment (bottom rows). For each comparison,

we report (i) the relative effect of the Public treatment, separately among healthier and less healthy subjects, and (ii) the interaction between (continuous) health and the Public (vs. other) treatment.

	**Study 1** (Healthier *n* = 375, Less healthy *n* = 367)	**Study 2** (Healthier *n* = 560, Less healthy *n* = 628)	**Studies 1 and 2** (Healthier *n* = 935, Less healthy *n* = 995)
**Public vs. Personal**			
Healthier	**b = 4.58, t = 2.34, d = .29,** p = .020	**b = 2.55, t = 1.75, d = .18,** p = .080	**b = 3.35, t = 2.86, d = .22,** p = .004
Less healthy	**b = 0.72, t = 0.33, d = .04,** p = .739	**b = 2.25, t = 1.41, d = .14,** p = .159	**b = 1.69, t = 1.31, d = .10,** p = .189
Interaction (with continuous health)	**b = 1.78, t = 1.26,** p = .208	**b = 1.33, t = 1.25,** p = .212	**b = 1.51, t = 1.77,** p = .076
**Public vs. Personal + Public**			
Healthier	**b = 6.07, t = 3.02, d = .38,** p = .003	**b = 1.52, t = 1.03, d = .11,** p = .304	**b = 3.32, t = 2.77, d = .22,** p = .006
Less healthy	**b = -1.95, t = -0.91, d = -.12,** p = .361	**b = 0.23, t = 0.15, d = .01,** p = .883	**b = -.58, t = -.45, d = -.04,** p = .651
Interaction (with continuous health)	**b = 3.39, t = 2.44,** p = .015	**b = 1.12, t = 1.09,** p = .278	**b = 2.00, t = 2.41,** p = .016

Table S5. Effects of the Public treatment as a function of subjective health. *Here we report effects of the Public treatment on prevention intentions, both relative to the Personal treatment and the Personal+Public treatment. For each treatment contrast, we report the effect of the Public (vs. other) treatment among healthier and less healthy individuals, as well as the interaction between our (continuous) subjective health measure and the Public (vs. other) treatment. We report these analyses of all subjects across the treatment conditions of (i) Study 1 (Column 1) (n = 742), (ii) Study 2 (Column 2) (n = 1188), and (ii) Studies 1 and 2 combined (Column 3) (n = 1930).*

Table S5 illustrates that (i) we found some evidence for an interaction between health and the effects of our Public treatment in Study 1, but (ii) we did not find meaningful support for this pattern in Study 2. Thus, our results do not provide robust support for the proposal that the Public treatment is especially effective among healthier individuals.

Table S5 also reveals that in analyses of only healthier individuals (i.e., the population that we planned to focus on in our Study 2 pre-registration), we continue to find evidence that the Public treatment was more effective than the Personal treatment, and no less effective than the Personal+Public treatment. Furthermore, the difference between the Public and Personal treatments observed in the pooled analysis of Studies 1 and 2 holds when accounting for multiple comparisons (p = .004, q_c = .026, q_s = .024), and when accounting for "peeking" (to maintain an actual type-I error rate of .05, it is necessary to evaluate statistical significance using an alpha threshold ranging from a "best-case scenario" of .049999 to a "worst-case scenario" of .0283).

4.7 Did the relative effectiveness of treatments depend on Time 1 prevention intentions in Study 3?

Here, we discuss the question of whether the relative effectiveness of our treatments in Study 3 varied as a function of Time 1 prevention intentions. To address this question, we shape our data to long format (with one observation for each prevention intention item) and use robust standard errors clustered on subject. Then, we conduct two sets of analyses. First, we predict

Time 2 prevention intentions as a function of treatment dummies, Time 1 prevention intentions, and their interactions. In the top rows of Table S6, we report the interaction for each pairwise comparison between treatments.

Second, we predict Time 2 prevention intentions as a function of treatment dummies and Time 1 prevention intentions, among observations for which the Time 1 prevention intention value is relatively lower (specifically, less than 80 on our 100-point scale; this pre-registered threshold is close to the median Time 1 prevention intention value, which is 79 both in Studies 3a-d and in Study 3d). In the bottom rows of Table S6, we compare each pair of treatments for this set of observations.

As illustrated by Table S6, across both analyses, we find no compelling evidence that the relative effectiveness of our treatments varied as a function of Time 1 prevention intentions.

	**Studies 3a-d (*n* = 3985)**	**Study 3d (*n* = 1773)**
	**Interaction between Time 1 intentions and pairwise comparison**	
**Public vs. Personal**	**b = -0.01, t = -0.51**, $p = .607$, $q_c = .939$	**b = -0.02, t = -0.97**, $p = .331$, $q_c = .701$
**Public vs. Personal+Public**	**b = -0.02, t = -1.16**, $p = .244$, $q_c = .568$	**b = 0.00, t = 0.07**, $p = .943$, $q_c = 1.000$
**Pesonal vs. Personal+Public**	**b = -0.01, t = -0.62**, $p = .537$, $q_c = .901$	**b = 0.02, t = 1.06**, $p = .291$, $q_c = .643$
	**Pairwise comparison if Time 1 intentions < 80**	
**Public vs. Personal**	**b = 0.41, t = 0.78, d = 0.01,** $p = .435$, $q_c = .820$	**b = 1.04, t = 1.36, d = 0.04,** $p = .176$, $q_c = .440$
**Public vs. Personal+Public**	**b = 1.01, t = 2.01, d = 0.04,** $p = .045$, $q_c = .129$	**b = 0.58, t = 0.76, d = 0.02,** $p = .448$, $q_c = .832$
**Pesonal vs. Personal+Public**	**b = 0.60, t = 1.20, d = 0.02,** $p = .232$, $q_c = .547$	**b = -0.46, t = -0.61, d = -0.02,** $p = .543$, $q_c = .905$

Table S6. Investigating the relative effectiveness of treatments as a function of Time 1 prevention intentions. *For each pairwise comparison, we report (i) the interaction between the relative effect of the treatment listed first and Time 1 prevention intentions on Time 2 prevention intentions (top rows), and (ii) the relative effect of the treatment listed first on Time 2 prevention intentions, controlling for Time 1 prevention intentions, among observations where the Time 1 prevention intention value is less than 80 (bottom rows). We report results both across Studies 3a-d, and in Study 3d only. We note that because our data are in long format, the standard deviation across observations is higher and Cohen's d values are lower, preventing a straightforward comparison to Cohen's d values from our wide format analyses.*

4.8 Analyses of "English check" in Studies 1-2 and attention checks in Study 3

In our Study 1-2 pre-registrations, we planned to conduct secondary analyses excluding subjects who appear not to speak English, on the basis of incorrect answers to a simple analogy question or incoherent responses to a simple free-response question. We coded answers to the simple analogy question (in a way that was blind to condition) for correct or near-correct answers (i.e., correct answers with typos/misspellings); across both studies (and all subjects), 6.99% of responses were incorrect. A visual scan of our data revealed that most subjects who

answered the analogy question incorrectly provided incoherent and/or irrelevant responses to the free-response question, while the vast majority of subjects who answered the analogy question correctly provided coherent and relevant answers. On this basis, we repeated our analyses excluding subjects who incorrectly answered the analogy question. We found that our results were unchanged qualitatively, but most patterns became a bit stronger. For brevity, we do not report these analyses; however, our “English check” data are available to interested readers.

In Study 3, we did not include this “English check”; however, in Studies 3a-c we instead asked subjects two simple attention checks (in which the question text instructed attentive subjects to select specific answer choices) and pre-registered secondary analyses investigating how our results vary for differing levels of attentiveness. We find no evidence that our key results vary significantly as a function of attentiveness; again, for brevity, we do not report these analyses but do make our attention check items available to interested readers.

4.9 Alternative choices about public and personal threat items in Study 4

In Study 4, we focused on certain specific items from Pennycook et al. 2020 that we felt mapped most closely onto our constructs of public versus personal threat (and our measures of these constructs from Studies 1-3). Here we demonstrate that the results are robust to different decisions about which items to use.

We begin by listing all items collected. Subjects rated their agreement (1 = Strongly disagree, 2 = Disagree, 3 = Somewhat disagree, 4 = Neither agree nor disagree, 5 = Somewhat agree, 6 = Agree, 7 = Strongly agree) with the following statements about COVID-19 risk:

1. The coronavirus poses a major threat to the public.
2. The coronavirus spread much faster than anyone anticipated.
3. People are not taking the coronavirus as seriously as they ought to.
4. There should be mass testing for the coronavirus.
5. The coronavirus is not as dangerous as people think. (R)
6. I think the situation with the coronavirus is overblown. (R)
7. Most people are not at risk of contracting the coronavirus. (R)
8. Very few people in the country are likely to actually get sick from the coronavirus. (R)

as well as these three items explicitly about themselves personally:

9. Because of my location, profession, and/or lifestyle, I am personally at a high risk of contracting the coronavirus.
10. Because of my age and/or pre-existing conditions, I am likely to have serious symptoms if I were to contract the coronavirus.
11. Because of my age and/or pre-existing conditions, I am likely to need hospitalization if I were to contract the coronavirus.

Our main text analysis uses item 1 as a measure of public threat and items 10 and 11 as a measure of personal threat. Table S7 shows the results using a variety of different combinations of items, revealing that our findings are robust to choices in measure construction. Below, we briefly discuss the logic behind (i) the set of combinations we chose to include in Table S7 and (ii) our choice of measures for our main text analysis.

In most analyses in Table S7, we include public threat measures constructed from some combination of items 1, 7, and 8, because these are the items that explicitly reference the public/population at large. We also saw items 1 and 8 as especially relevant because, like our measures from Studies 1-3, they focus on threat to the public and the consequences (rather than likelihood) of contracting coronavirus. And we chose to specifically use item 1 in our main text analyses because of its high face validity and because, like our measures from Studies 1-3, it is straightforwardly worded (i.e., not reverse coded).

Turning to personal threat, items 9-11 all explicitly reference the individual. We also saw items 10 and 11 as especially relevant because, like our measures from Studies 1-3, they focus on the consequences (rather than likelihood) of contracting coronavirus. We thus chose to use them in our primary analysis as our measure of personal threat.

	Public=1 v Personal=10,11			Public=1,8 v Personal=10,11			Public=1,7,8 v Personal=10,11		
**Personal threat**	**0.282*** **	**0.191*** **	**0.179*** **	**0.282*** **	**0.197*** **	**0.187*** **	**0.282*** **	**0.209*** **	**0.193*** **
	(0.0351)	(0.0336)	(0.0397)	(0.0351)	(0.0338)	(0.0401)	(0.0351)	(0.0339)	(0.0404)
**Public threat**	**0.418*** **	**0.371*** **	**0.355*** **	**0.401*** **	**0.353*** **	**0.342*** **	**0.379*** **	**0.333*** **	**0.322*** **
	(0.0333)	(0.0336)	(0.0354)	(0.0335)	(0.0338)	(0.0370)	(0.0339)	(0.0339)	(0.0373)
**Public vs.**	t(745)=3.24	F(1,745)=11.56	F(1,709)=8.58	t(745)=2.81	F(1,745)=8.66	F(1,709)=6.40	t(745)=2.23	F(1,745)=5.49	F(1,709)=4.36
**Personal**	p=0.001	p<0.001	p=0.004	p=0.005	p=0.003	p=0.012	p=0.026	p=0.019	p=0.037

	Public=1 v Personal=9,10,11			Public=1,8 v Personal=9,10,11			Public=1,7,8 v Personal=9,10,11		
**Personal threat**	**0.303*** **	**0.200*** **	**0.187*** **	**0.303*** **	**0.204*** **	**0.194*** **	**0.303*** **	**0.218*** **	**0.204*** **
	(0.0349)	(0.0339)	(0.0381)	(0.0349)	(0.0343)	(0.0385)	(0.0349)	(0.0342)	(0.0386)
**Public threat**	**0.418*** **	**0.361*** **	**0.346*** **	**0.401*** **	**0.342*** **	**0.332*** **	**0.379*** **	**0.321*** **	**0.313*** **
	(0.0333)	(0.0339)	(0.0356)	(0.0335)	(0.0343)	(0.0372)	(0.0339)	(0.0342)	(0.0374)
**Public vs.**	t(745)=2.84	F(1,745)=8.77	F(1,709)=7.14	t(745)=2.41	F(1,745)=6.29	F(1,709)=5.12	f(745)=1.81	F(1,745)=3.59	F(1,709)=3.21
**Personal**	p=0.005	p=0.003	p=0.008	p=0.016	p=0.012	p=0.024	p=0.07	p=0.059	p=0.074

	Public=1-8 v Personal=9,10,11		
**Personal threat**	**0.486*** **	**0.434*** **	**0.446*** **
	(0.0320)	(0.0332)	(0.0370)
**Public threat**	**0.303*** **	**0.164*** **	**0.138*** **
	(0.0349)	(0.0332)	(0.0377)
**Public vs.**	t(745)=4.78	F(1,745)=24.93	F(1,709)=25.22
**Personal**	p<0.001	p<0.001	p<0.001

Standard errors in parentheses
*** p<0.01, ** p<0.05, * p<0.1

Table S7. Study 4's observation that public threat is a stronger predictor of prevention intentions is robust to choices in measure construction. *Shown are the results from regressions predicting prevention intentions as a function of the composition thread variables collected in Study 4 using various different formulations of public versus personal threat. Each box heading reports the item(s) used to form the public and personal threat scales. We then show, in each box, results from (i) a set of separate regression models for each threat variable (left column within each box) and (ii) multiple regression models using both threat variables, both without (center column within each box) and with (right column within each box) controls for age, gender, education, race, income, and political party affiliation. All coefficients are standardized coefficients, with standard errors for each coefficient in parentheses. For each model, we also report results from a test comparing the public versus personal threat coefficient.*

5. Discussion of pre-registered analysis plans

We independently pre-registered Studies 1 (https://aspredicted.org/blind.php?x=mb9t3e), 2 (https://aspredicted.org/blind.php?x=w8jk9m), 3a (https://aspredicted.org/blind.php?x=2bh82k), 3b (https://aspredicted.org/blind.php?x=78tn97), 3c (https://aspredicted.org/blind.php?x=bq9xd6), and 3d (https://aspredicted.org/blind.php?x=qv9rz3). We adhered closely to our pre-registered analysis plans, with a few exceptions. The substantive exceptions are noted in the main text where relevant, but here we provide a comprehensive list of all deviations. We also note that Studies 4 and 5, as well as our analyses in Studies 1-3 investigating the associations between our personal and public threat variables and prevention intentions, were not pre-registered. However, the fact that our personal and public threat analyses replicate across studies provides evidence against false positives.

5.1 Studies 1-2

First, in both of our Study 1 and 2 pre-registrations, we planned only to report results among all subjects, and not to explore the order in which we measured our dependent variables versus potential mediators. However, after completing both studies, we discovered an unexpected interaction between condition and order. Thus, to confirm the robustness of our results, for analyses of our dependent variables, we report results (i) among all subjects, and (ii) among subjects for whom we measured our dependent variables *before* measuring our potential mediators.

Second, in our Study 1 pre-registration, we planned to focus equally on both of our dependent variables (i.e., prevention intentions and social distancing intentions). However, as mentioned in main text, in Study 1 the prevention intentions variable produced stronger evidence for treatment effects and interesting differences between treatments, and thus in Study 2 we chose to focus on replicating these results. For this reason, we focus our paper on prevention intentions. Specifically, while we report primary analyses of social distancing intentions (i.e., treatment effects relative to control, and comparisons between treatment effects), we do not report analyses of the relationships between social distancing intentions and our individual difference variables or candidate mediators, or heterogeneity in treatment effects on intentions to avoid individual social behaviors.

Third, in our Study 1 pre-registration, we planned to compare all pairs of treatments to each other. However, given our pattern of results, when analyzing prevention intentions we chose to focus on the comparison of the Public treatment to each of the other two treatments, and thus do not compare the Personal treatment to the Public+Personal treatment. We pre-registered this plan before running Study 2.

Fourth, in our Study 1 pre-registration, we planned, as a secondary analysis, to explore treatment effects on intentions to engage in individual prevention behaviors, and to avoid individual social activities. Additionally, we noted that we were in particular concerned about ceiling effects, and thus would repeat our primary analyses looking only to the prevention behaviors and social activities for which baseline responses were the relatively lowest (i.e., furthest from ceiling). We did, in fact, explore individual prevention behaviors (see Figure S1), and in the main text we also report an analysis of the overall social distancing item included in our composite measure of prevention intentions. But because we did find treatment effects on prevention intentions (i.e., there was not a ceiling effect) and we found no significant heterogeneity across individual behaviors, we did not repeat our primary analyses looking only

to behaviors furthest from ceiling. We note, however, that Figure S1 sorts individual behaviors by average baseline responses (i.e., distance from ceiling) for interested readers. (As noted above, we also did not explore intentions to avoid individual social activities, given our primary focus on our prevention intentions dependent variable).

Fifth, in our Study 2 pre-registration, we planned for our primary analyses to focus specifically on healthier individuals (defined as individuals reporting subjective health above the Study 1 median). As noted in the main text and the "moderation by subjective health" section of this SI, this decision reflected that, in Study 1, we found evidence suggesting that healthier individuals show relatively larger Public treatment effects. However, evidence for an interaction between health and our Public treatment effects was weaker in Study 2 than in Study 1. Thus, despite the fact that this interaction pattern makes theoretical sense, we did not feel confident focusing on it in our primary analyses, and instead chose to focus primarily on main effects among all subjects. We note, however, that as shown in the "moderation by subjective health" section of this SI, analyses of healthy individuals also support our key findings from Studies 1-2.

Relatedly, in our Study 2 pre-registration, we also planned, as a secondary analyses, to (i) repeat our primary analyses among subjects reporting zero pre-existing health conditions, and (ii) test for interactions between pre-existing health conditions and the Public treatment. But because we chose not to focus extensively on moderation by health, we do not report these analyses.

Finally, our pre-registrations for Studies 1-2 did not plan to compute q-values to correct for multiple comparisons. We also only pre-registered each of our two studies individually and did not pre-register a plan to pool data from both studies.

5.2 Study 3

First, in our Study 3 pre-registrations, we planned to analyze our data in long format (with one observation for each prevention intention item). However, as described in the main text, for consistency with our approach from Studies 1-2, we instead primarily analyze data in wide format (computing composite prevention intentions across our 10 items). We do, however, analyze the data in long format as planned when investigating whether the relative effectiveness of treatments is influenced by Time 1 prevention intentions. We note that our conclusions from all analyses are qualitatively unchanged when analyzing in wide versus long format.

Second, in our Study 3 pre-registrations, we planned to explore moderation by our individual difference variables. However, given that we do not find any overall differences in the effectiveness of our treatments, for brevity we do not report moderation analyses.

Third, in our Study 3 pre-registrations, we simply planned to investigate absolute ratings of our two threat variables (i.e., the perceived public and personal threat of coronavirus) for the purpose of comparison to our earlier studies. However, we nonetheless investigate whether these variables showed the same patterns as in our earlier studies (both with respect to whether they were influenced by our manipulations, and their associations with prevention intentions).

Fourth, while each of our Study 3b-d pre-registrations planned to investigate the effectiveness of each of our treatments (by comparing Time 2 prevention intentions to Time 1 prevention intentions), we accidentally omitted this analysis from our Study 3a pre-registration. We nonetheless performed this analysis using data from Study 3a.

Finally, we only pre-registered each of Studies 3a-d individually and did not pre-register a plan to pool data across studies.

6. Experimental materials

Here, we show the stimuli used in our studies. Additionally, all experimental materials are available here.

6.1 Written text from treatments

In the main text, we report the sections of the written text used in our treatments that varied across treatments. Here, we report the sections that were constant across treatments.

Studies 1-2, before the section that varied across treatments:

> Coronavirus disease 2019 (COVID-19) is a respiratory illness that can spread from person to person. The virus that causes COVID-19 is a novel coronavirus that was first identified during an investigation into an outbreak in Wuhan, China. Because COVID-19 is a novel virus, there is no immunity in the community yet. There is also no vaccine for COVID-19.
>
> COVID-19 is currently spreading rapidly through the US. As of today, there are at least 1,701 confirmed cases, and this number is likely a major underestimate given that testing in the US has been extremely limited. The number of cases is growing exponentially. According to one projection by the Center for Disease Control (CDC), between 160 million and 214 million people in the U.S. could be infected over the course of the epidemic. As many as 200,000 to 1.7 million people could die. And, the calculations based on the CDC's scenarios suggested, 2.4 million to 21 million people in the U.S. could require hospitalization, potentially crushing the nation's medical system, which has only about 925,000 staffed hospital beds. Fewer than a tenth of those are for people who are critically ill.
>
> COVID-19 is much worse than the ordinary flu. The flu has a death rate of around 0.1% of infections. Globally, about 3.4 percent of reported COVID-19 cases have died. Furthermore, experts think COVID-19 is more contagious than the ordinary flu. And people can spread COVID-19 before experiencing any symptoms.

Note that in Study 2, we updated the case count information to read "As of Sunday night, there are now over 3,000 confirmed cases".

Studies 1-2, after the section that varied across treatments:

> It is recommended that you practice good personal hygiene (wash your hands, avoid shaking hands or hugging others, avoid touching your face, and cover your mouth when you cough or sneeze), stay home if you are even a little bit sick, practice social distancing (by staying home as much as possible and avoiding close contact with others), and prepare by purchasing food reserves, medication, and cleaning supplies.

Study 3, before the section that varied across treatments:

> Coronavirus disease 2019 (COVID-19) is spreading rapidly through the United States. As of Thursday night, there are now over 680,000 confirmed cases and over 35,000 deaths in the U.S. One factor that makes COVID-19 so difficult to contain is that people can spread the virus before experiencing any symptoms.
>
> There is a long road ahead before life can return to normal. Most experts expect that a vaccine will not be ready for 12-18 months. And until a vaccine is ready, it will remain possible for new outbreaks to emerge.

Note that in Studies 3b-d, we updated the case and death count information to 800,000 and 45,000 (Study 3b), 850,000 and 47,000 (Study 3c), and 1,064,000 and 61,600 (Study 3d).

Study 3, after the section that varied across treatments:

> Specifically, it is important to engage in social distancing by minimizing physical interactions, wearing a mask when outside the house, and staying at least 6 ft away from others. It is also still important to practice good hygiene by washing hands frequently and trying not to touch your face. These actions are likely to remain important even after government "stay at home" orders end.
>
> Furthermore, it may also be important for people to allow the government to access their health data and track their movements and the people who they are interacting with (i.e., "contract tracing") and/or regularly get tested for the virus.

6.2 Dependent variables

Studies 1-2 and 4 measure of prevention intentions:

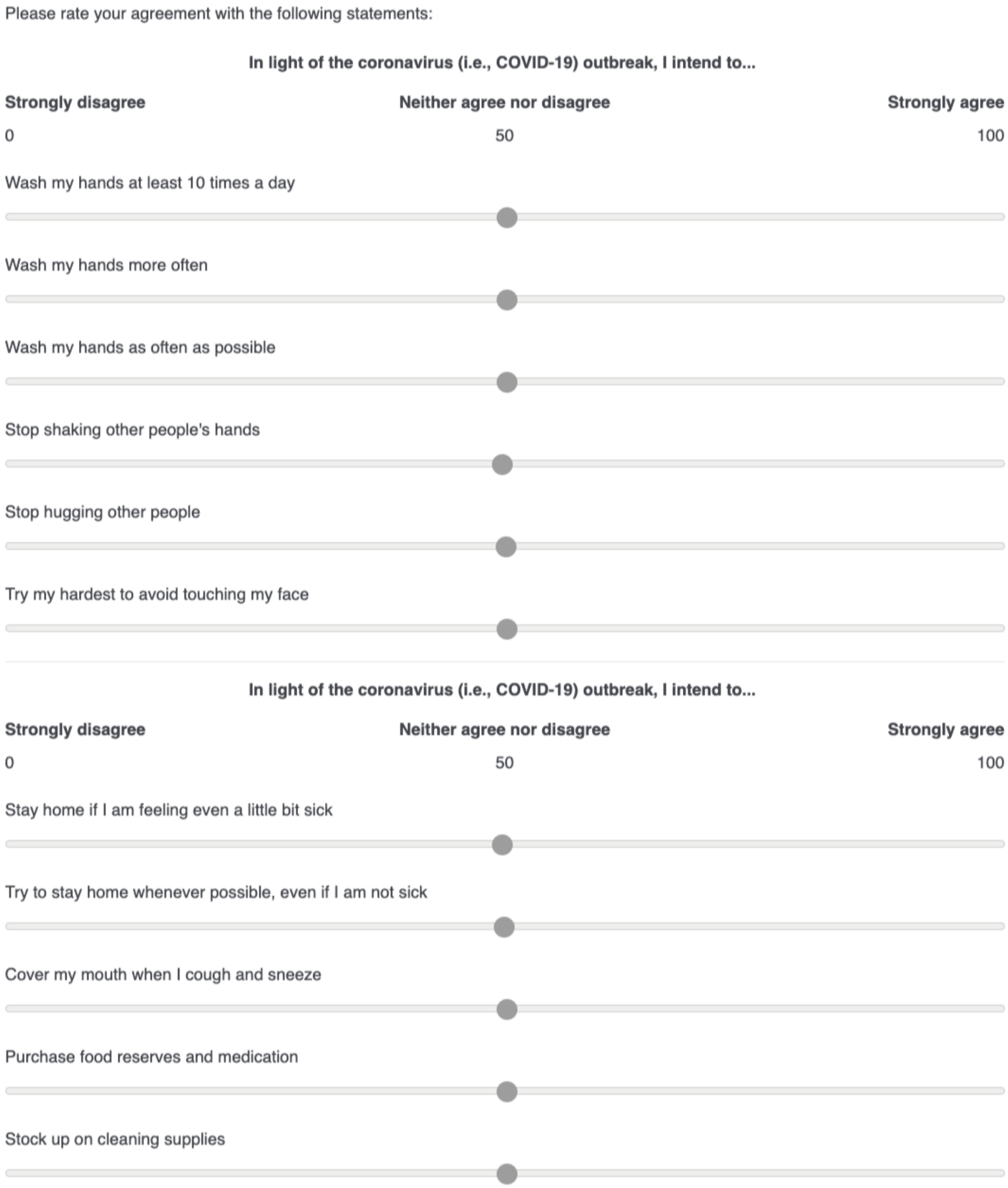
Please rate your agreement with the following statements:

In light of the coronavirus (i.e., COVID-19) outbreak, I intend to...

**Strongly disagree**	**Neither agree nor disagree**	**Strongly agree**
0	50	100

Wash my hands at least 10 times a day

Wash my hands more often

Wash my hands as often as possible

Stop shaking other people's hands

Stop hugging other people

Try my hardest to avoid touching my face

In light of the coronavirus (i.e., COVID-19) outbreak, I intend to...

**Strongly disagree**	**Neither agree nor disagree**	**Strongly agree**
0	50	100

Stay home if I am feeling even a little bit sick

Try to stay home whenever possible, even if I am not sick

Cover my mouth when I cough and sneeze

Purchase food reserves and medication

Stock up on cleaning supplies

Note that these items were presented in the fixed order shown here.

Study 1 measure of social distancing intentions:

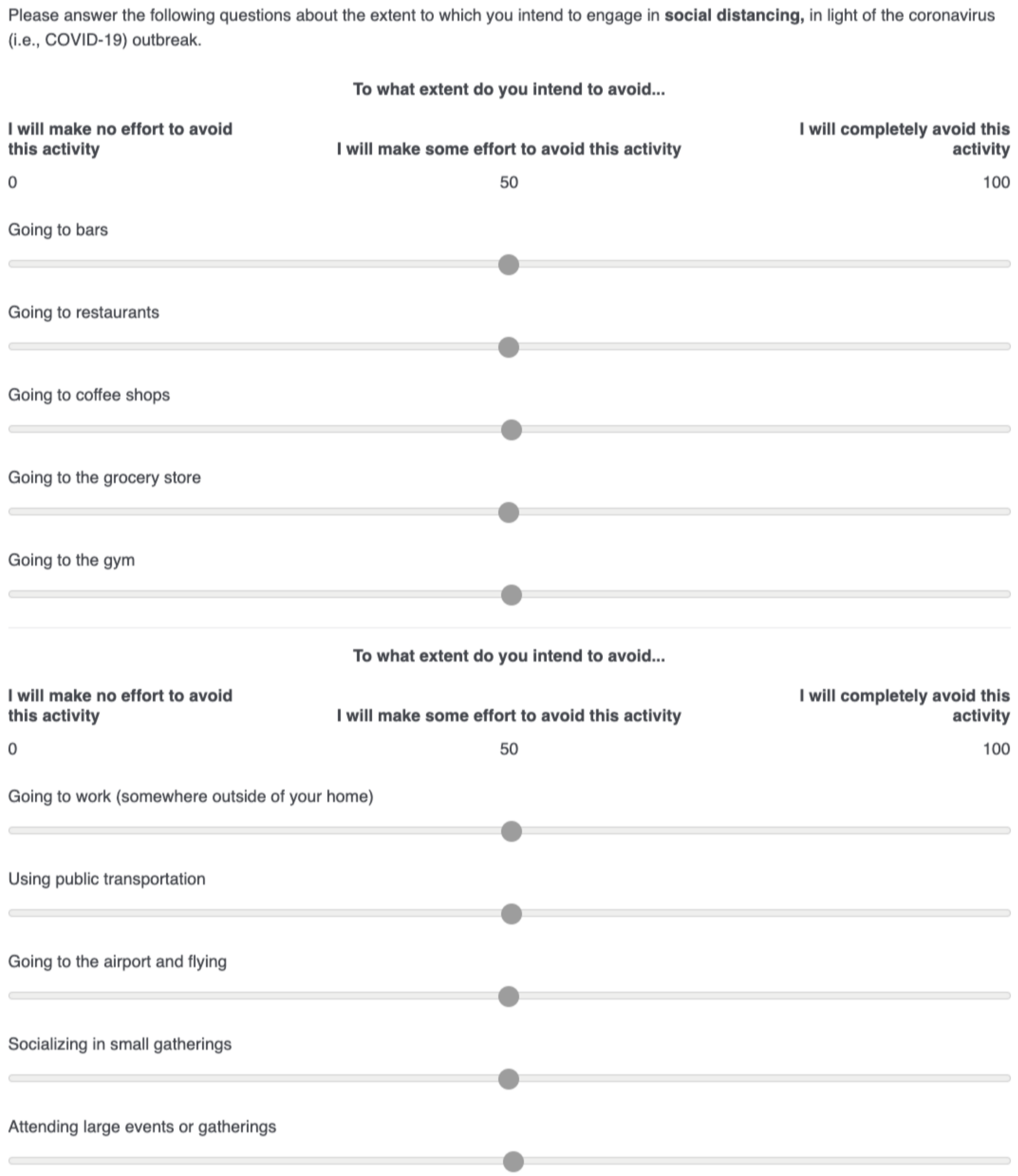
Please answer the following questions about the extent to which you intend to engage in **social distancing,** in light of the coronavirus (i.e., COVID-19) outbreak.

To what extent do you intend to avoid...

I will make no effort to avoid this activity	I will make some effort to avoid this activity	I will completely avoid this activity
0	50	100

Going to bars

Going to restaurants

Going to coffee shops

Going to the grocery store

Going to the gym

To what extent do you intend to avoid...

I will make no effort to avoid this activity	I will make some effort to avoid this activity	I will completely avoid this activity
0	50	100

Going to work (somewhere outside of your home)

Using public transportation

Going to the airport and flying

Socializing in small gatherings

Attending large events or gatherings

Note that these items were presented in the fixed order shown here.

On the previous page, we asked you about your intentions to avoid engaging in a variety of behaviors.

If coronavirus were NOT a concern, which of these these behaviors would you at least sometimes engage in? **Please check all that apply.**

- [ ] Going to bars
- [ ] Going to restaurants
- [ ] Going to coffee shops
- [ ] Going to the grocery store
- [ ] Going to the gym
- [ ] Going to work (somewhere outside of your home)
- [ ] Using public transportation
- [ ] Going to the airport and flying
- [ ] Socializing in small gatherings
- [ ] Attending large events or gathering

Note that these items were presented in the fixed order shown here.

Study 3 measure of prevention intentions:

Please rate your agreement with the following statements, which pertain to the COVID-19 pandemic.

Even after official stay-at-home orders end, I intend to...

Strongly disagree	Neither agree nor disagree	Strongly agree
0	50	100

Wear a mask when I leave the house for the foreseeable future

Wash my hands as much as possible for the foreseeable future

If relevant, allow the government to regularly test me for COVID-19

Avoid crowded indoor or outdoor spaces for the foreseeable future

Try my hardest to avoid touching my face for the foreseeable future

Limit my physical interaction with others when possible for the foreseeable future

Refrain from visiting anyone who is sick at the hospital, even if they are a close family member and even if they are dying

Even if I think I have contracted and recovered from COVID-19, remain vigilant unless I have a confirmed positive test (either for the virus during infection, or antibodies after infection)

Completely avoid any unnecessary physical contact with others (e.g., hugging or handshakes) for the foreseeable future

If relevant, allow the government to track my health data, movements, and/or the people I interact with

Note that these items were presented in random order, and this screenshot shows our Time 1 measure of prevention intentions. Our Time 2 measure was identical, except (i) the introductory statement at the top was replaced with "Please now rate your agreement with the following statements a second time", and (ii) the order was re-randomized.

6.3 Perceived public and personal threat of coronavirus

Study 1 measure of perceived personal threat:

To what extent are you afraid **for yourself** of contracting coronavirus (i.e., COVID-19)? In other words, to what extent do you fear the **personal repercussions for you** that would follow from contracting coronavirus?

**Not at all**	**To a moderate extent**	**To an enormous extent**
0	50	100

How large of a **personal threat** do you think coronavirus (i.e., COVID-19) poses to you?

**No threat at all**	**A moderate threat**	**An enormous threat**
0	50	100

Study 1 measure of perceived public threat:

How large of a **societal threat** do you think coronavirus (i.e., COVID-19) poses to your community?

**No threat at all**	**A moderate threat**	**An enormous threat**
0	50	100

To what extent are you afraid **for your community** of contracting and spreading coronavirus (i.e., COVID-19)? In other words, to what extent do you fear the **collective repercussions for society** that would follow from contracting and spreading coronavirus?

**Not at all**	**To a moderate extent**	**To an enormous extent**
0	50	100

Note that these two pages were measured in random order, but the order of questions on each page was fixed in the order shown. Due to a programming error, this meant that the question starting with "to what extent are you afraid" was shown first when measuring *personal* threat, and second when measuring *public* threat.

Study 2 measure of perceived personal threat:

To what extent are you afraid of contracting coronavirus (i.e. COVID-19) because of the consequences **for you personally?**

**Not at all**	**To a moderate extent**	**To an enormous extent**
0	50	100

How large of a **personal threat** do you think coronavirus (i.e., COVID-19) poses to you?

**No threat at all**	**A moderate threat**	**An enormous threat**
0	50	100

Study 2 measure of perceived public threat:

How large of a **collective threat** do you think coronavirus (i.e., COVID-19) poses to your community?

**No threat at all**	**A moderate threat**	**An enormous threat**
0	50	100

To what extent are you afraid of contracting and spreading coronavirus (i.e. COVID-19) because of the consequences **for your community?**

**Not at all**	**To a moderate extent**	**To an enormous extent**
0	50	100

Note that these two pages were measured in random order, but the order of questions on each page was fixed in the order shown. Due to a programming error, this meant that the question starting with "to what extent are you afraid" was shown first when measuring *personal* threat, and second when measuring *public* threat.

Study 3 used our Study 2 measures but corrected the aforementioned programming error by randomized the order of questions on each page.

For threat measures in Study 4, see SI Section 4.10.

6.4 Individual difference variables

Studies 1-2 initial set of questions:

Please answer the following questions about yourself.

Age:

Gender

- Male
- Female

Highest level of education completed:

- Less than a high school degree
- High School Diploma
- Vocational Training
- Attended College
- Bachelor's Degree
- Graduate Degree
- Unknown

What is your zip code?

Overall, taking into account any health conditions that you have, how would you evaluate your health?

Terrible	Very bad	Bad	Moderate	Good	Very good	Excellent
○	○	○	○	○	○	○

Please consider the following list of health conditions:

Cardiovascular diseases, diabetes, hepatitis B, chronic obstructive pulmonary disease, chronic kidney diseases, and cancer.

How many of these conditions do you have?

- 0
- 1
- 2
- 3
- 4
- 5 or more

Please choose the category that describes the total amount of income you earned in 2019. Consider all forms of income, including salaries, tips, interest and dividend payments, scholarship support, student loans, parental support, social security, alimony, and child support, and others.

- Under $5,000
- $5,000-$10,000
- $10,001-$15,000
- $15,001-$25,000
- $25,001-$35,000
- $35,001-$50,000
- $50,001-$65,000
- $65,001-$80,000
- $80,001-$100,000
- Over $100,000

Which US political party do you identify with more strongly?

1-Strongly Democrat	2	3	4-Neutral	5	6	7-Strongly Republican
○	○	○	○	○	○	○

Politically, how conservative are you in terms of social issues?

1 - Very liberal	2	3	4	5	6	7 - Very conservative
○	○	○	○	○	○	○

Politically, how conservative are you in terms of economic issues?

1 - Very liberal	2	3	4	5	6	7 - Very conservative
○	○	○	○	○	○	○

Prior to taking this survey, much information have you been exposed to about COVID-9?

**Almost none**	**A moderate amount**	**An enormous amount**
0	50	100

Note that these items were presented in the fixed order shown here. Note also that to compute a "college degree" dummy from our categorical education variable, we coded the data as follows: 0 = "less than a high school degree", "high school diploma", "vocational training", "attended college", or "unknown"; 1 = "bachelor's degree" or "graduate degree".

Study 3 included most of these questions, with a few exceptions outlined in SOM Section 1. It also additionally included the two below questions:

Please specify your race. (*Choose one or more categories)*

- ☐ White/Caucasian (Anglo/Euro) American
- ☐ Black or African American
- ☐ Asian or Asian American
- ☐ American Indian or Alaska Native
- ☐ Native Hawaiian or other Pacific Islander
- ☐ Hispanic/Latino
- ☐ Multicultural

Prior to taking this survey, approximately how many previous surveys about COVID-19 have you taken?

Note that our analyses of race included a series of dummies for each racial category.

Cognitive Reflection Task used in all studies except 3d:

Please do your best to answer the following questions as accurately as possible.

The ages of Mark and Adam add up to 28 years total. Mark is 20 years older than Adam. How many years old is Adam?

If it takes 10 second for 10 printer to print out 10 pages of paper, how many seconds will it take 50 printers to print out 50 pages of paper?

On a loaf of bread, there is a patch of mold. Every day, the patch doubles in size. If it takes 40 days for the patch to cover the entire loaf of bread, how many days would it take for the patch to cover half of the loaf of bread?

How many of the last 3 questions do you think you answered correctly?

Note that each of these four questions was presented on a separate page, in the fixed order shown here. We did not analyze responses to the final question (about the number of questions answered correctly). Correct answers to the first three questions: 4, 10, 39.

English check used in Studies 1-2:

Please, now answer a final set of questions.

Dog is to puppy as cat is to _____

Please write 3 sentences about what you've done with your day so far, or what you plan to do for the rest of the day.

Attention checks used in Study 3a-c:

When a big news story breaks, people often go online to get up-to-the-minute details on what is going on. We want to know which websites people trust to get this information. We also want to know if people are paying attention to the question. Please ignore the question and select FoxNews.com and NBC.com as your two answers.

When there is a big news story, which is the one news website you would visit first? (Please choose only one):

- New York Times website
- Huffington Post
- CNN.com
- FoxNews.com
- Google News
- Yahoo! News
- NBC.com
- USA Today website
- Other

In the grid below, you will see a series of statements. Please tell us whether you agree or disagree with each statement.

	Agree strongly	Agree	Neither agree nor disagree	Disagree	Disagree strongly
People convicted of murder should be given the death penalty.					
Please click the "neither agree nor disagree" response.					
Gays and lesbians should have the right to legally marry.					
World War I came after World War II.					
In order to reduce the budget deficit, the federal government should raise taxes on people that make more than $250,000 per year.					
The Affordable Care Act passed by Congress in 2010 should be repealed.					
The government should require all electricity power plants to significantly reduce their greenhouse gas emissions even if it might increase electricity bills a few dollars a month.					

Note that we presented the first attention check after measuring Time 2 prevention intentions (but before measuring the perceived public and personal threat of coronavirus) and we presented the second attention check after measuring all individual difference questions except for the CRT.

6.5 Field experiment materials

The following are the three versions of the materials included in Covid Act Now (CAN)'s daily newsletter. The first version featured our *Personal* treatment in the header, and was sent to subscribers with last names that began with the letters A through I on December 21, 2021 with the email subject line "A new variant spreading in the U.K.". The header read:

Add your phone to your state's [exposure notification system] to receive alerts when you have been in close contact with someone who later tests positive for COVID. Once you activate the system, you'll need to keep your bluetooth on in order to receive alerts. Your privacy is protected as your identity is not known and your location is not tracked. Participating in the exposure system is one of the most effective actions you can take at the moment. It lets you know if you may have COVID, which helps you obtain treatment more quickly.

The remaining content of that day's newsletter is available at the following url: http://createsend.com/t/t-DCD20D92AB1E0AE12540EF23F30FEDED

The second version featured our *Public* treatment in the header, and was sent to subscribers with last names that began with the letters J through Q on December 22, 2021 with the email subject line "California hospitals overwhelmed". The header read:

Add your phone to your state's [exposure notification system] to receive alerts when you have been in close contact with someone who later tests positive for COVID. Once you activate the system, you'll need to keep your bluetooth on in order to receive alerts. Your privacy is protected as your identity is not known and your location is not tracked. Participating in the exposure system is one of the most effective actions you can take at the moment. It lets you know if you may have COVID, which prevents you from spreading COVID to more people.

The remaining content of that day's newsletter is available at the following url: http://createsend.com/t/t-7BABFD2072E69B4E2540EF23F30FEDED

The third version featured our *Personal+Public* treatment in the header, and was sent to subscribers with last names that began with the letters R through Z on December 23, 2021 with the email subject line "100 million more doses". It read:

Add your phone to your state's [exposure notification system] to receive alerts when you have been in close contact with someone who later tests positive for COVID. Once you activate the system, you'll need to keep your bluetooth on in order to receive alerts. Your privacy is protected as your identity is not known and your location is not tracked. Participating in the exposure system is one of the most effective actions you can take at the moment. It lets you know if you may have COVID, which helps you obtain treatment more quickly and prevents you from spreading COVID to more people.

The remaining content of that day's newsletter is available at the following url: http://createsend.com/t/t-CF9B1E0EB93DF96C2540EF23F30FEDED
